# Temporal Trends and Sociodemographic Differences in Telemedicine Utilization, 2019–2024

**DOI:** 10.1007/s11606-025-09964-y

**Published:** 2026-02-11

**Authors:** Bingyu Zhang, Lu Li, Yiwen Lu, Jingchuan Guo, Jiang Bian, John B. Salmon, Robert L. Stetson, Michael A. Horst, Srinivas K. Sridhara, Mitchell D. Schnall, Kevin B. Mahoney, David A. Asch, Yong Chen

**Affiliations:** 1 Center for Health AI and Synthesis of Evidence (CHASE), University of Pennsylvania, Philadelphia, PA USA; 2 The Graduate Group in Applied Mathematics and Computational Science, School of Arts and Sciences, University of Pennsylvania, Philadelphia, PA USA; 3 Department of Pharmaceutical Outcomes and Policy, University of Florida College of Pharmacy, Gainesville, FL USA; 4 Center for Drug Evaluation and Safety, University of Florida, Gainesville, FL USA; 5 Department of Biostatistics and Health Data Science, School of Medicine, Indiana University, Indianapolis, IN USA; 6 Regenstrief Institute, Indianapolis, IN USA; 7 University of Pennsylvania Health System, Philadelphia, PA USA; 8 Department of Radiology, Hospital of the University of Pennsylvania, Philadelphia, PA USA; 9 Office of the Chief Executive Officer, University of Pennsylvania Health System, Philadelphia, PA USA; 10 Department of Medicine, Perelman School of Medicine, University of Pennsylvania, Philadelphia, PA USA; 11 Penn Center for Health Incentives and Behavioral Economics, University of Pennsylvania, Philadelphia, PA USA; 12 Wharton School, University of Pennsylvania, Philadelphia, PA USA; 13 Leonard Davis Institute of Health Economics, University of Pennsylvania, Philadelphia, PA USA; 14 Division of General Internal Medicine, University of Pennsylvania, Philadelphia, PA USA; 15 Department of Biostatistics, Epidemiology, and Informatics, University of Pennsylvania Perelman School of Medicine, Philadelphia, PA USA; 16 Penn Medicine Center for Evidence-Based Practice (CEP), Philadelphia, PA USA; 17 Penn Institute for Biomedical Informatics (IBI), Philadelphia, PA USA

**Keywords:** electronic health records, patient characteristics, telemedicine, temporal trends

## Abstract

**Background:**

Telemedicine usage surged during the COVID-19 pandemic, shaping how patients access healthcare services. Its sustained role in post-pandemic healthcare may uncover long-term trends and variations in utilization.

**Objective:**

To characterize telemedicine utilization from 2019 to 2024 and identify patient characteristics associated with telemedicine use.

**Design and Participants:**

This retrospective cohort study analyzed outpatient visits across five hospitals within the University of Pennsylvania Health System (Penn Medicine) from January 1, 2019, to September 30, 2024.

**Main Measures:**

The primary outcome was whether each outpatient encounter was conducted via telemedicine (vs in-person). We used multivariable logistic regression clustering on patients to assess associations between telemedicine use and patient- and encounter-level characteristics, including demographics, insurance, patient portal use, income, clinical comorbidity, distance from care, provider specialty, encounter type, hospital index, and visit year.

**Key Results:**

The study included 46,149,734 visits among 2,248,341 patients. Telemedicine surged from 1% of visits pre-pandemic to 17% in April 2020, stabilized at 8–13% through late 2020, and remained 4–6% from 2022 to 2024. Telemedicine use was lower among older adults (aOR 0.67 for ages 40–64; 0.47 for ≥ 65 vs. < 40 years), males (aOR 0.90), and new visits (aOR 0.46). Higher use was observed among unmarried (aOR 1.10), patient portal users (aOR 1.44), patients with fewer comorbidities, those living ≥ 15 miles from care (aOR 1.42 vs. < 5 miles), lower-income (< $50,000 aOR 1.06 vs. $50,000-$100,000), and primary care (aOR 1.23 vs. specialty care). Telemedicine use was lower among Non-Hispanic Black (aOR 0.88), Hispanic (aOR 0.94), and Asian (aOR 0.82) patients compared to Non-Hispanic White patients. Patterns differed by clinical condition, with disproportionately higher use among White patients with mental health disorders.

**Conclusions:**

Telemedicine use persists post-pandemic but reflects differences in access by age, race/ethnicity, socioeconomic status, and prior engagement with the patient portal. Targeted policies are needed to ensure equitable telemedicine adoption and accessibility for all patients.

**Supplementary Information:**

The online version contains supplementary material available at 10.1007/s11606-025-09964-y.

## Introduction

During the COVID-19 pandemic, most large health systems rapidly expanded their telemedicine capabilities to ensure continuity of patient care. Telemedicine was used for forward triage, automated patient screening, and integrating COVID-19 testing into care workflows.^1–4^ At the University of Pennsylvania Health System (Penn Medicine), this included tripling capacity for remote monitoring, incorporating virtual consultations at a newly opened inpatient facility, and leveraging digital tools to enhance patient safety and minimize in-hospital risks.^5^

Several studies have documented the rise in telemedicine encounters during the early and later phases of the pandemic, highlighting its adoption, peak utilization, and subsequent decline as in-person services resumed.^6–13^ However, most of these studies are limited to short-term trends, specific populations, or discrete time points within the pandemic, leaving gaps in understanding telemedicine’s long-term integration into routine care. Evidence on sociodemographic differences in telemedicine adoption is also mixed.^14–19^ For example, findings by race and ethnicity are inconsistent: some studies report no differences,^14,16^ while others find higher or lower utilization among minority groups compared with White patients.^15,17,19^ Insurance-related patterns are also unclear, with some studies reporting lower use among publicly insured patients (Medicare or Medicaid)^15,19^ and others finding no difference.^17,18^ Few investigations have examined these patterns across multiple years.

In addition, relatively little is known about how telemedicine use varies across provider specialties and clinical conditions, despite that organizational context and condition-specific workflows shape the sustainability of virtual care. Prior work has examined primary care or behavioral health in isolation,^13,17^ with limited comparisons across specialties and diagnoses.

This study aimed to address these gaps using a large electronic health records (EHR)-derived dataset from Penn Medicine, a large-scale health system with a patient population reflective of U.S. demographic diversity, encompassing more than 46 million outpatient visits from 2019 to 2024. By spanning the pre-pandemic, pandemic, and post-pandemic periods, this analysis provides one of the longest continuous perspectives on telemedicine adoption to date. We extended prior work in three important ways: (1) By providing a multi-year view that captures pre-pandemic, pandemic, and post-pandemic telemedicine use, (2) By examining how sociodemographic differences persisted or evolved over this extended period between telemedicine and in-person visit users, and (3) By characterizing variation across provider specialties and specific medical conditions. Together, these contributions provide new insights into how telemedicine has been integrated into routine care and identify opportunities for more targeted dissemination strategies to enhance equity and condition-specific adaptation.

## Methods

### Data and Study Population

This study used EHR data from five hospitals within Penn Medicine, including the Hospital of the University of Pennsylvania (HUP), Penn Presbyterian Medical Center (PPMC), Pennsylvania Hospital (PAH), Chester County Hospital (CCH), and Penn Medicine Princeton Medical Center (PMC). Data were collected from January 1, 2019, to September 30, 2024.

The Penn Medicine EHR is a comprehensive, longitudinal data resource that supports clinical research across a wide spectrum of health conditions and treatments. It includes real-world data from inpatient and outpatient encounters across multiple hospitals and affiliated practices, covering over 6.5 million unique patients and more than 50 million clinical encounters. This study included data from one EHR instance across the system representing care provided in the Philadelphia region.

We included outpatient encounters designated as face-to-face (F2F) visits in the EHR data, which include both in-person encounters and telemedicine video visits. Emergency department visits or inpatient visits were excluded. Encounters not classified as F2F, such as telephone-only, laboratory, radiology, orders-only, or administrative notes, were also excluded to ensure a meaningful comparison across visit types. A complete list of included visit types is provided in the Supplementary Materials eTable 1.

### Primary Outcomes and Patient Characteristics

The primary outcome was telemedicine use at the visit level, defined as whether each encounter was conducted via telemedicine (vs in-person), and modeled as a binary variable in regression analyses. Patient characteristics considered in the analysis included: age (< 40, 40–64, ≥ 65 years),^20^ sex (Male, Female), race/ethnicity (self-identified; Hispanic, Non-Hispanic Black, Non-Hispanic White, Non-Hispanic Asian, Other/Unknown), insurance type (Commercial, Medicaid, Medicare, Self-Pay/Other),^15,21^ user of the patient portal (Yes, No), marital status (Married, Unmarried), median household income (estimated from zip code-level census data;^22^ < $50,000, $50,000 to $100,000, ≥ $100,000), and Charlson Comorbidity Index scores^23^ (< 3, ≥ 3). Encounter-level characteristics included encounter category (New patient visit, Return patient visit, Other/Unknown), provider specialty (Primary care, Specialty care, Unknown), hospital index (five Penn Medicine hospitals), distance from home to the place of service (estimated from the distance between patient home address and place of service zip codes;^24^ < 5 miles, 5–15 miles, ≥ 15 miles), and visit year (2019–2024).

### Statistical Analysis

We described temporal trends in telemedicine usage from 2019 to 2024. We used multivariable logistic regression to estimate the association between patient- and encounter-level characteristics and the likelihood that a visit was conducted via telemedicine (vs in-person). To account for correlation among repeated encounters contributed by the same patient, models were clustered on patient ID and estimated using generalized estimating equations with robust standard errors. The reference group was generally chosen as the category with the largest sample size or based on conventions in prior literature.

To examine variations in telemedicine utilization for specific conditions, we conducted additional analyses focused on selected chronic conditions, including diabetes, mental disorders, sleep disorders, heart failure, chronic obstructive pulmonary disease (COPD), coronary artery disease (CAD), and gastrointestinal (GI) disorders. Conditions of interest were identified using ICD-10-CM diagnosis codes recorded in the EHR. A complete list of codes used for each condition category is provided in Supplementary eTable 3. These conditions were chosen because they are common and well-suited for either in-person or telemedicine care. We repeated the above descriptive and regression models within these disease-specific subpopulations to investigate the temporal patterns and identify factors that may drive telemedicine usage uniquely in these groups. Two-sided P < 0.05 was considered statistically significant.

All analyses were performed using R version 4.3.1. This study was reviewed and classified as exempt by the University of Pennsylvania Institutional Review Board. This study followed the Strengthening the Reporting of Observational Studies in Epidemiology (STROBE) reporting guideline.

## Results

### Cohort Identification

We identified 46,149,734 outpatient encounters among 2,248,341 patients within Penn Medicine between January 1, 2019, and September 30, 2024. Of these, 2,269,344 (4.9%) were completed via telemedicine, and 43,880,390 (95.1%) occurred in person. Among all visits, 62.7% were women; 61.4% were Non-Hispanic White, 22.6% were Non-Hispanic Black, 4.8% were Hispanic, 4.7% were Non-Hispanic Asian; 78.7% were users of the patient portal.

Patients who completed telemedicine visits were more likely to be women, younger adults, patient portal users, return patients, unmarried, and with lower Charlson Comorbidity Index scores. A detailed comparison of baseline characteristics between telemedicine and in-person visits is summarized in Table 1, with additional patient-level characteristics in eTable 4 and hospital-level characteristics in eTable 12.

**Table 1:** Baseline Characteristics Between Telemedicine and In-Person Visits among Outpatient Encounters from 2019 to 2024

	Telemedicine Visits (N = 2,269,344)	In-person Visits (N = 43,880,390)	Overall (N = 46,149,734)	Telemedicine Utilization Rate
**Visit Year, no. (%)**				
2019	48763 (2.1)	7421019 (16.9)	7469782 (16.2)	0.65%
2020	700158 (30.9)	6455362 (14.7)	7155520 (15.5)	9.78%
2021	500002 (22.0)	7505506 (17.1)	8005508 (17.3)	6.25%
2022	395005 (17.4)	7735083 (17.6)	8130088 (17.6)	4.86%
2023	350273 (15.4)	8165043 (18.6)	8515316 (18.5)	4.11%
2024	275143 (12.1)	6598377 (15.0)	6873520 (14.9)	4.00%
**Hospital, no. (%)**				
CCH	104373 (4.6)	4122384 (9.4)	4226757 (9.2)	2.47%
HUP	1311811 (57.8)	20267793 (46.2)	21579604 (46.8)	6.08%
PMC	293015 (12.9)	5981315 (13.6)	6274330 (13.6)	4.67%
PAH	277269 (12.2)	6295894 (14.3)	6573163 (14.2)	4.22%
PPMC	282876 (12.5)	7213004 (16.4)	7495880 (16.2)	3.77%
**Age Range, no. (%)**				
< 40 years	830246 (36.6)	10561066 (24.1)	11391312 (24.7)	7.29%
40–64 years	877811 (38.7)	17008227 (38.8)	17886038 (38.8)	4.91%
≥ 65 years	561287 (24.7)	16311097 (37.2)	16872384 (36.6)	3.33%
**Sex, no. (%)**				
Female	1510314 (66.6)	27423206 (62.5)	28933520 (62.7)	5.22%
Male	759030 (33.4)	16457184 (37.5)	17216214 (37.3)	4.41%
**Race/Ethnicity, no. (%)**				
Non-Hispanic White	1417677 (62.5)	26895385 (61.3)	28313062 (61.4)	5.01%
Non-Hispanic Black	478386 (21.1)	9948154 (22.7)	10426540 (22.6)	4.59%
Hispanic	116809 (5.1)	2083501 (4.7)	2200310 (4.8)	5.31%
Asian	97128 (4.3)	2082864 (4.7)	2179992 (4.7)	4.46%
Other/Unknown	159344 (7.0)	2870486 (6.5)	3029830 (6.6)	5.26%
**Insurance Plan, no. (%)**				
Commercial	1164593 (51.3)	18903127 (43.1)	20067720 (43.5)	5.80%
Medicaid	291334 (12.8)	5084662 (11.6)	5375996 (11.6)	5.42%
Medicare	595933 (26.3)	15443489 (35.2)	16039422 (34.8)	3.72%
Self-Pay/Other	217484 (9.6)	4449112 (10.1)	4666596 (10.1)	4.66%
**MyPennMedicine User, no. (%)**				
No	302287 (13.3)	9507494 (21.7)	9809781 (21.3)	3.08%
Yes	1967057 (86.7)	34372896 (78.3)	36339953 (78.7)	5.41%
**Marital Status, no. (%)**				
Married	1094117 (48.2)	22795968 (52.0)	23890085 (51.8)	4.58%
Unmarried	1175227 (51.8)	21084422 (48.0)	22259649 (48.2)	5.28%
**Encounter Category, no. (%)**				
Return Patient Visit	1818743 (80.1)	23247661 (53.0)	25066404 (54.3)	7.26%
New Patient Visit	214514 (9.5)	4309405 (9.8)	4523919 (9.8)	4.74%
Other/Unknown	236087 (10.4)	16323324 (37.2)	16559411 (35.9)	1.43%
**Median household income, no. (%)**				
< $50,000	426673 (18.8)	8294546 (18.9)	8721219 (18.9)	4.89%
$50,000 to $100,000	1116982 (49.2)	20645586 (47.0)	21762568 (47.2)	4.63%
≥ $100,000	725689 (32.0)	14940258 (34.0)	15665947 (33.9)	5.13%
**Charlson Comorbidity Index score, no. (%)**				
< 3	1407067 (62.0)	23568167 (53.7)	24975234 (54.1)	5.63%
≥ 3	862277 (38.0)	20312223 (46.3)	21174500 (45.9)	4.07%
**Distance from Home to Place of Service, no. (%)**				
< 5 miles	773178 (34.1)	16018615 (36.5)	16791793 (36.4)	4.60%
5 to 15 miles	773465 (34.1)	16475670 (37.5)	17249135 (37.4)	4.48%
≥ 15 miles	722701 (31.8)	11386105 (25.9)	12108806 (26.2)	5.97%
**Provider Specialty, no. (%)**				
Primary Care	247559 (10.9)	2720825 (6.2)	2968384 (6.4)	8.34%
Specialty Care	1950526 (86.0)	25409781 (57.9)	27360307 (59.3)	7.13%
Unknown	71259 (3.1)	15749784 (35.9)	15821043 (34.3)	0.45%

### Temporal Trends in Telemedicine Utilization

**Figure 1:**
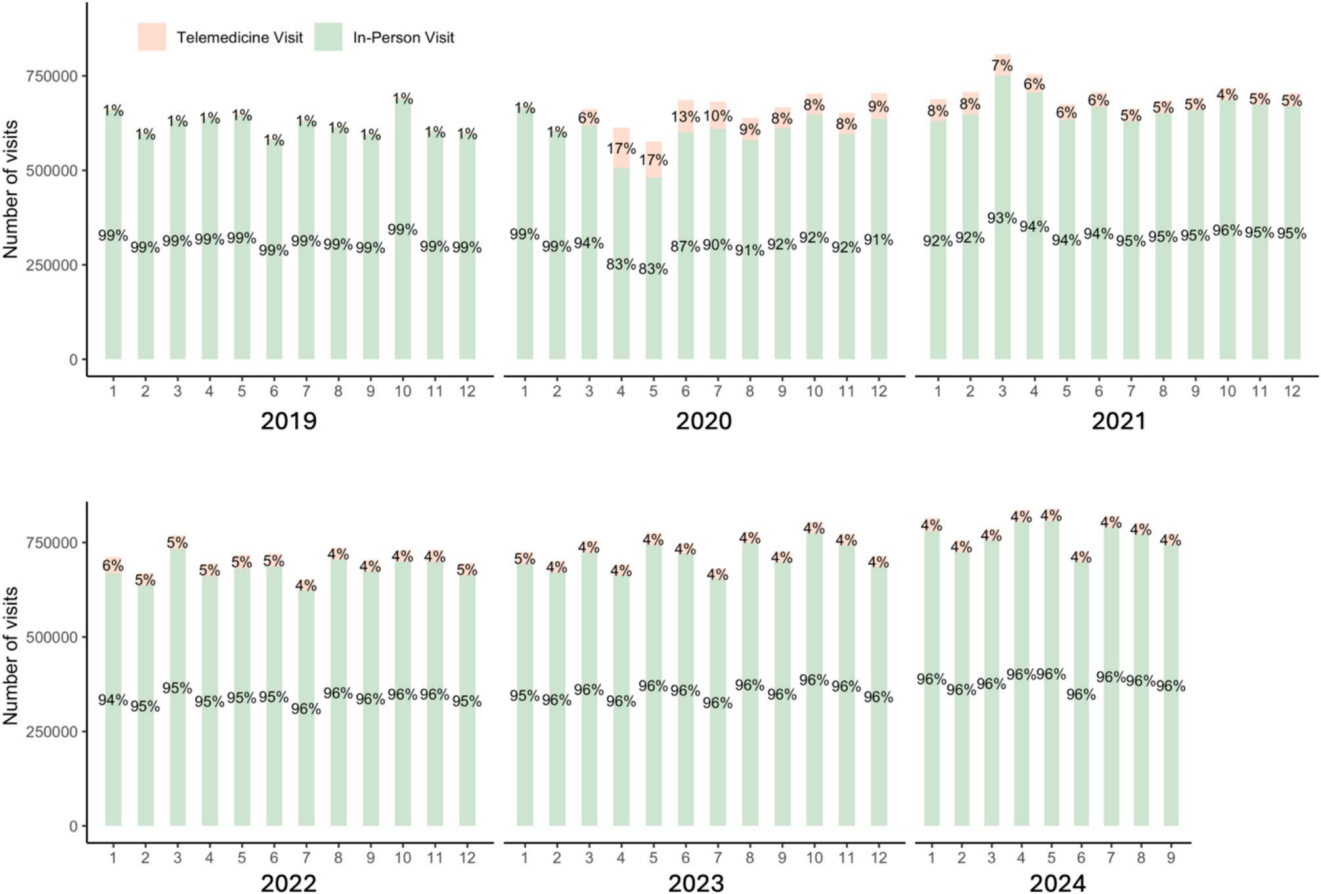
Telemedicine utilization rate by calendar time, monthly from 2019 to 2024.

Figure 1 illustrates the monthly number of telemedicine and in-person visits from 2019 to 2024. From 2019 to early 2020, telemedicine usage was minimal (around 1%). With the onset of COVID-19 in March 2020, telemedicine visits surged from 1 to 17% by April and fluctuated between 8–13% for the rest of the year. Utilization declined in 2021 and stabilized around 4–6% from 2022 onward, suggesting a continued but reduced role for telemedicine as in-person visits regained prominence.

**Figure 2:**
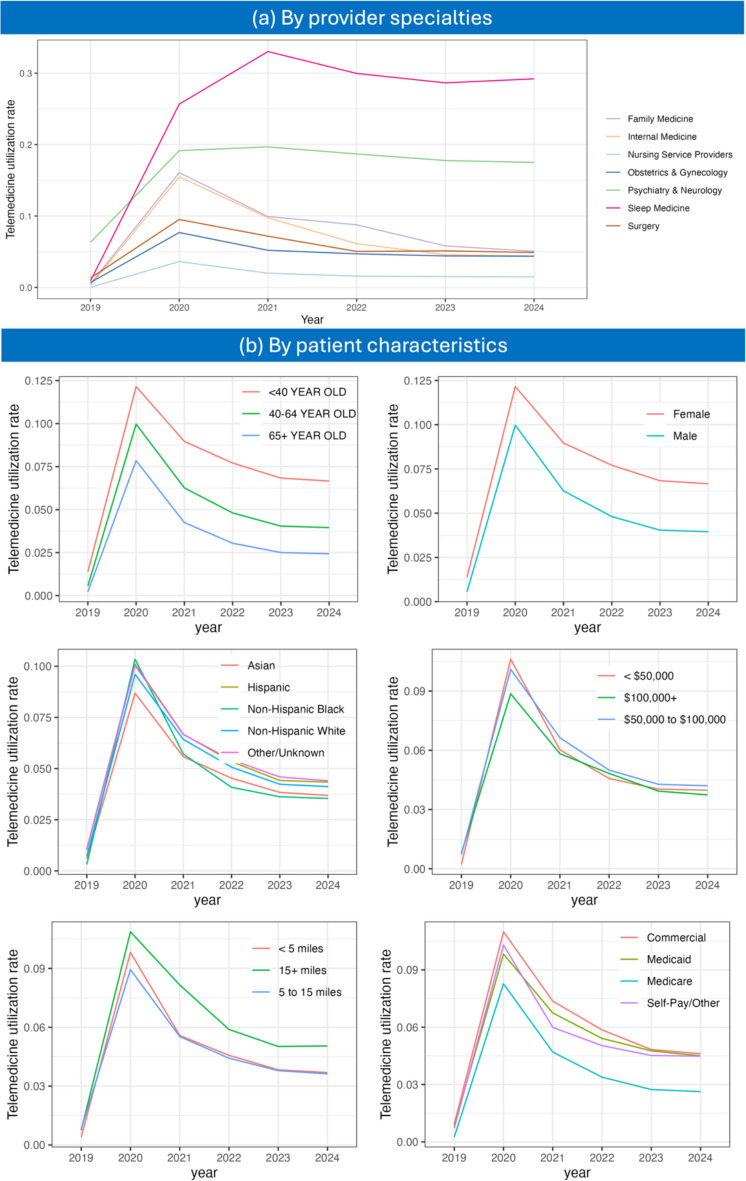
Temporal patterns of telemedicine visit rates categorized by provider specialties and patient characteristics.

Figure 2a shows the telemedicine utilization rates categorized by provider specialties. Sleep medicine, and psychiatry & neurology saw the highest telemedicine use, peaking at 20–35% in 2021 and remaining relatively high into 2024. Family medicine, internal medicine, surgery, and obstetrics & gynecology peaked at around 10–15% and declined to 5%. Nursing services had the lowest telemedicine rates, underscoring the continued need for in-person care in those fields. Only specialties with high prevalence were reported; the full list is available in the Supplementary Materials eTable 2.

Figure 2b illustrates temporal patterns of telemedicine utilization stratified by various characteristics. Younger individuals (< 40 years) had the highest telemedicine adoption rates, while older adults (65+ years) consistently reported lower utilization. Females demonstrated higher telemedicine usage across all time points compared to males. Patients with household incomes exceeding $100,000 had a lower rate of telemedicine use. Non-Hispanic White patients reported slightly greater telemedicine usage than other racial and ethnic groups. Patients residing more than 15 miles from healthcare facilities showed higher telemedicine adoption rates than those living closer. Patients with commercial insurance had the highest telemedicine usage throughout the study period, whereas those with Medicare demonstrated lower rates.

### Factors Associated with Telemedicine Use

Compared with patients younger than 40 years, older patients were less likely to receive care via telemedicine (40–64 years: aOR, 0.67 [95% CI, 0.67–0.67]; ≥ 65 years: aOR, 0.47 [95% CI, 0.47–0.47]). Compared with Non-Hispanic White patients, Asian (aOR, 0.82, 95% CI, 0.82–0.83), Hispanic (aOR, 0.94, 95% CI, 0.94–0.95), and Non-Hispanic Black (aOR, 0.88, 95% CI, 0.88–0.89) patients were associated with less telemedicine use. Male (aOR: 0.90, 95% CI, 0.90–0.91) and new patient visits (aOR: 0.46, 95% CI, 0.46–0.47) were associated with less telemedicine use. Unmarried patients (aOR, 1.10, 95% CI, 1.10–1.11) and patient portal users (aOR, 1.44, 95% CI, 1.43–1.45) were more likely to use telemedicine. Patients with higher Charlson Comorbidity Index scores (aOR, 0.88 [95% CI, 0.88–0.88]) were less likely to use telemedicine. A median household income greater than $100,000 was associated with slightly less telemedicine use compared with an income of $50,000 to $100,000 (aOR, 0.91, 95% CI, 0.91–0.92). Longer distance from home to the place of service is associated with more telemedicine usage (5 to 15 miles: aOR, 1.04, 95% CI, 1.03–1.04; ≥ 15 miles: aOR, 1.42, 95% CI, 1.41–1.42). Visits with a primary care provider had higher telemedicine utilization compared to specialty care (aOR, 1.23, 95% CI, 1.22–1.24). Table 2 summarizes the results from the regression model.

**Table 2:** Multivariable Logistic Regression Model Associations with Telemedicine and In-Person Visits among Outpatient Encounters from 2019 to 2024

Characteristics	Adjusted Odds Ratio (95% CI)
**Visit Year**	
2019	1.00 [Reference]
2020	17.86 (17.69–18.03)
2021	11.42 (11.32–11.53)
2022	8.10 (8.02–8.18)
2023	6.57 (6.51–6.63)
2024	6.21 (6.15–6.27)
**Hospital**	
HUP	1.00 [Reference]
CCH	0.45 (0.44–0.45)
PMC	1.17 (1.17–1.18)
PAH	0.57 (0.57–0.58)
PPMC	0.58 (0.58–0.59)
**Age Range**	
< 40 years	1.00 [Reference]
40–64 years	0.67 (0.67–0.67)
≥ 65 years	0.47 (0.47–0.47)
Sex	
Female	1.00 [Reference]
Male	0.90 (0.90–0.91)
**Race/Ethnicity**	
Non-Hispanic White	1.00 [Reference]
Asian	0.82 (0.82–0.83)
Hispanic	0.94 (0.94–0.95)
Non-Hispanic Black	0.88 (0.88–0.89)
Other/Unknown	0.96 (0.96–0.97)
**Insurance Plan**	
Commercial	1.00 [Reference]
Medicaid	0.99 (0.98–0.99)
Medicare	1.07 (1.07–1.08)
Self-Pay/Other	1.14 (1.14–1.15)
**MyPennMedicine User**	
No	1.00 [Reference]
Yes	1.44 (1.43–1.45)
**Marital Status**	
Married	1.00 [Reference]
Unmarried	1.10 (1.10–1.11)
**Encounter Category**	
Return Patient Visit	1.00 [Reference]
New Patient Visit	0.46 (0.46–0.47)
Other/Unknown	0.27 (0.26–0.27)
**Median household income**	
< $50,000	1.06 (1.05–1.06)
$50,000 to $100,000	1.00 [Reference]
≥ $100,000	0.91 (0.91–0.92)
**Charlson Comorbidity Index score**	
< 3	1.00 [Reference]
≥ 3	0.88 (0.88–0.88)
**Distance from Home to Place of Service**	
< 5 miles	1.00 [Reference]
5 to 15 miles	1.04 (1.03–1.04)
≥ 15 miles	1.42 (1.41–1.42)
**Provider Specialty**	
Primary Care	1.23 (1.22–1.24)
Specialty Care	1.00 [Reference]
Unknown	0.06 (0.06–0.06)

### Visits Categorized by Different Health Conditions

**Figure 3:**
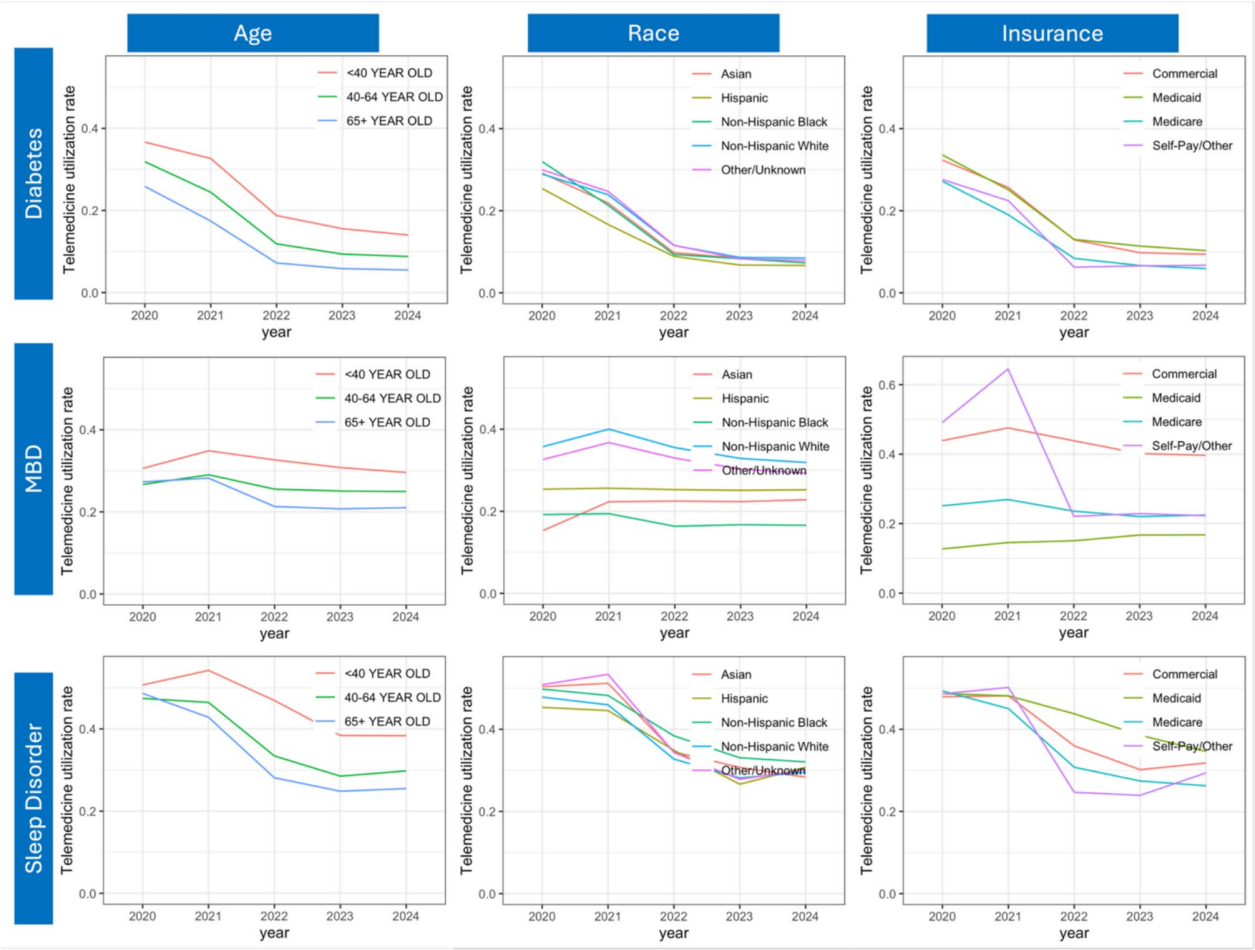
Temporal patterns of characteristics for visits of three health conditions, including diabetes, mental, behavioral, and neurodevelopmental disorders (MBD), and sleep disorders, stratified by age, race/ethnicity, and insurance type.

Figure 3 illustrates detailed temporal patterns of telemedicine utilization across visits of three health conditions, including diabetes, mental, behavioral, and neurodevelopmental disorders (MBD), and sleep disorders, stratified by age, race/ethnicity, and insurance type. Comprehensive temporal patterns for seven health conditions across multiple patient and encounter characteristics are provided in eFigures 1–7.

Across these diagnoses, the trends in age, sex, and distance from home to the place of service remain consistent. Younger patients, female patients, and patients living further from the place of service are more likely to use telemedicine. Utilization rates for these conditions declined after 2021 but remained above pre-pandemic levels, likely reflecting the convenience of remote monitoring and follow-ups for chronic conditions that require routine management.

Telemedicine utilization for patients with diabetes peaked during the pandemic, particularly among younger patients. Sleep disorders demonstrated sustained high telemedicine utilization post-pandemic. For patients with heart failure and COPD, telemedicine use was more modest. Both CAD and GI disorders followed similar patterns of low-to-moderate telemedicine use.

Multivariable regression results for visits within each health condition are presented in eTables 5–11. These models confirmed the descriptive findings shown in Fig. 3. Building on these models, interaction analyses highlighted additional differences within MBD-related visits (eTables 14–15). Specifically, Non-Hispanic White patients had higher odds of telemedicine use compared with other racial/ethnic groups, and self-pay/other insurance types showed higher odds of telemedicine use in 2020 and 2021, though this difference diminished after 2022. This trend may be attributed to policy changes expanding coverage for MBD-related telemedicine visits under commercial insurance plans.

## Discussion

This study highlights the trends in telemedicine and in-person visits from Penn Medicine from 2019 to 2024, revealing significant shifts in care delivery during the COVID-19 pandemic. Our findings show a rapid surge in telemedicine use during the early months of the pandemic, followed by stabilization at lower but sustained levels as in-person care resumed. The initial spike reflects the healthcare system’s swift adaptation to pandemic restrictions, while the sustained utilization suggests that telemedicine has become a complementary mode of care delivery, particularly for follow-up visits and consultations that do not require physical examinations.

Our analysis highlighted key demographic and socioeconomic differences in telemedicine use. Younger patients (< 40 years) consistently exhibited higher adoption, whereas older adults (65 + years) showed lower utilization rates, possibly due to barriers such as technology access.^25^ In sensitivity analyses using finer categories (eTable 13), patients aged ≥ 75 were the least likely to use telemedicine, confirming especially low uptake among the oldest adults.^26^ Additionally, female patients, individuals with lower household incomes, and patient portal users were more likely to use telemedicine.

The breakdown by specialty also aligns with findings from prior research, which reported sustained telemedicine use in psychiatry and primary care but lower adoption in procedural specialties such as surgery. These patterns underscore the importance of tailoring telemedicine strategies to the needs of specific patient populations and specialties. For example, incorporating hybrid care models that balance telemedicine with in-person visits could address the needs of patients with chronic conditions requiring both routine monitoring and hands-on evaluations.^27^

System-level factors within Penn Medicine also influenced telemedicine adoption. Not all outpatient visits had equal opportunity for telehealth encounters, as internal scheduling algorithms directed certain visit types toward in-person care, even for routine chronic condition follow-ups. These algorithms, which evolved throughout the study period, likely contributed to variations in telemedicine utilization and may have created an upper limit on the number of visits eligible for telehealth. Additionally, provider and clinic-level biases played a role, as some providers actively embraced telehealth, while others remained resistant, even for visit types well-suited for remote care. Understanding these structural and provider-driven influences is crucial for future telemedicine optimization.

Our analysis accounted for such contextual differences by including hospital fixed effects. This approach was appropriate given the small number of hospitals and their heterogeneity, which spans downtown tertiary referral centers, urban community hospitals, and suburban sites. Prior studies similarly highlight that system-level factors, including differences in infrastructure investment and scheduling practices, influence telemedicine adoption.^12^ By incorporating hospital fixed effects, our models accounted for these system-level influences and strengthened the robustness of our findings.

The condition-specific analysis revealed that telemedicine use varied by the clinical nature of each condition. Mental health and sleep disorders maintained consistently high utilization rates, reflecting their suitability for remote care delivery. Conditions such as heart failure and COPD, which often require physical assessments and complex management, showed moderate telemedicine adoption, with rates declining post-pandemic. Chronic conditions like diabetes require sustained use for routine monitoring and follow-ups, whereas procedural or diagnostic-heavy conditions, such as CAD and GI disorders, consistently rely more on in-person care. These variations emphasize the need to align telemedicine practices with the specific demands of different clinical conditions.

Insurance coverage and regulatory policies played a pivotal role in telemedicine uptake. Prior to the COVID-19 pandemic, many insurers did not routinely reimburse e-visits, and health systems often lacked established billing pathways. The federal public health emergency and subsequent policy changes rapidly expanded coverage, allowing telemedicine encounters to be reimbursed on par with in-person visits. These changes facilitated the surge in utilization we observed during 2020. Importantly, telemedicine reimbursement remains tied to regulatory restrictions, including requirements that patients be physically located in the same state as their provider. Such policies may differentially affect access across populations and geographic regions, reinforcing the need for sustained policy attention to ensure equitable telemedicine adoption beyond the pandemic period.

Limitations of this study include that the data are specific to Penn Medicine and may not generalize to other healthcare settings with different patient populations and telemedicine infrastructures. Race and ethnicity were self-identified in the Penn Medicine EHR. Also, while we analyzed trends over a multi-year period, telemedicine adoption was influenced not only by patient preference but also by institutional scheduling rules and provider-driven biases, which were not fully captured in our models. Future research should explore the impact of these system-level constraints on telemedicine availability and uptake.

## Supplementary Information

Below is the link to the electronic supplementary material.

ESM 1 (DOCX 33.9 KB)

ESM 2 (DOCX 2.65 MB)

## Data Availability

The results reported in this study are based on detailed individual-level patient data. The datasets generated and analyzed are not publicly available due to the University of Pennsylvania’s privacy and confidentiality requirements.

## Declarations

### Preprint Information

This manuscript has been posted on medRxiv: 10.1101/2025.03.26.25324648v1.
