# Supplementary material for: Temporal Trends and Sociodemographic Differences in Telemedicine Utilization, 2019–2024
Source: J Gen Intern Med. 2026 Feb 11;41(9):2407–15. doi: 10.1007/s11606-025-09964-y (PMC13304034; doi:10.1007/s11606-025-09964-y)

**Supplementary Materials to “Temporal Trends and Sociodemographic Differences in Telemedicine Utilization, 2019-2024”**

Table of Contents

[eTable 1. Face-to-face list of encounter types 3](#_Toc209734658)

[eTable 2. Complete list of provider specialties 4](#_Toc209734659)

[eTable 3. Study variables 5](#_Toc209734660)

[eTable 4. Patient-level baseline characteristics 7](#_Toc209734661)

[eTable 5. Odds ratios for diabetes visits 8](#_Toc209734662)

[eTable 6. Odds ratios for MBD visits 10](#_Toc209734663)

[eTable 7. Odds ratios for sleep awake disorder visits 12](#_Toc209734664)

[eTable 8. Odds ratios for heart failure visits 14](#_Toc209734665)

[eTable 9. Odds ratios for COPD visits 16](#_Toc209734666)

[eTable 10. Odds ratios for CAD visits 18](#_Toc209734667)

[eTable 11. Odds ratios for GI visits 20](#_Toc209734668)

[eTable 12. Baseline characteristics for different health systems 22](#_Toc209734669)

[eTable 13. Odds ratios with a finer age category 23](#_Toc209734670)

[eTable 14. Odds ratios with the interaction term of MBD and race/ethnicity 25](#_Toc209734671)

[eTable 15. Odds ratios with the interaction term of visit year and insurance within MBD visits 27](#_Toc209734672)

[eTable 16. Crude odds ratios 29](#_Toc209734673)

[eFigure 1. Temporal patterns for diabetes visits 31](#_Toc209734674)

[eFigure 2. Temporal patterns for MBD visits 32](#_Toc209734675)

[eFigure 3. Temporal patterns for sleep awake disorder visits 33](#_Toc209734676)

[eFigure 4. Temporal patterns for heart failure visits 34](#_Toc209734677)

[eFigure 5. Temporal patterns for COPD visits 35](#_Toc209734678)

[eFigure 6. Temporal patterns for CAD visits 36](#_Toc209734679)

[eFigure 7. Temporal patterns for GI visits 37](#_Toc209734680)

# eTable 1. Face-to-face list of encounter types

| **Encounter Type** | **Encounter Type Name** |
| --- | --- |
| 2 | Walk-In |
| 3 | Hospital Encounter |
| 11 | Research Encounter |
| 50 | Appointment |
| 76 | Telemedicine |
| 81 | Ophth Exam |
| 91 | Home Care Visit |
| 101 | Office Visit |
| 108 | Immunization |
| 120 | Endoscopy |
| 200 | Allied Health Visit |
| 436 | PT/OT/ST |
| 438 | OBGYN Visit |
| 441 | In Home Primary Care Encounter |
| 449 | PEC Visit |
| 450 | OBGYN SURGERY |
| 452 | Out of Office Visit |
| 600 | General Surgery |
| 604 | Anticoagulation Therapy |
| 610 | Infusion Visit |
| 1003 | Procedure Visit |
| 2523 | Hospice F2F Visit |
| 32002 | Care Management |
| 32003 | Virtual Visit |
| 32006 | Psych Office Visit |
| 32009 | Psych Allied Health |
| 32012 | Procedure |
| 32013 | Allied Health Visit (Non-Chargeable) |
| 32030 | Telehealth |
| 32032 | Psych Allied Health (Non-Chargeable) |
| 304113 | Palliative Home Care Visit |
| 304114 | CCBH Scheduled |
| 304117 | Telemedicine (Non-Chargeable) |
| 1650000002 | TXP Evaluation |

# eTable 2. Complete list of provider specialties

The provider specialties were categorized according to <https://taxonomy.nucc.org/>.

| **Provider Specialty Clusters** |
| --- |
| Anesthesiology |
| Behavioral Health & Social Service Providers |
| Chiropractic Providers |
| Critical Care |
| Dental Providers |
| Dermatology |
| Dietary & Nutritional Service Providers |
| Emergency Medicine |
| Eye and Vision Services Providers |
| Family Medicine |
| Hospice and Palliative Medicine |
| Hospitalist |
| Hospitals |
| Internal Medicine |
| Medical Genetics |
| Nuclear Medicine |
| Nursing Service Providers |
| Obstetrics & Gynecology |
| Ophthalmology |
| Other Service Providers |
| Pathology |
| Pediatrics |
| Pharmacy Service Providers |
| Physical Medicine & Rehabilitation |
| Physician Assistants & Advanced Practice Nursing Providers |
| Podiatric Medicine & Surgery Service Providers |
| Preventive Medicine |
| Psychiatry & Neurology |
| Radiology |
| Residential Treatment Facilities |
| Respiratory, Developmental, Rehabilitative and Restorative Service Providers |
| Sleep Medicine |
| Speech, Language and Hearing Service Providers |
| Sports Medicine |
| Surgery |
| Urology |

# eTable 3. Study variables

| Variable | Functional form | Values | Detail |
| --- | --- | --- | --- |
| Outcome |  |  |  |
| Visit type | Indicator | Telemedicine/In-person visit | Based on the encounter type in eTable 1. The values “Telemedicine”, “Virtual visit”, “Telehealth”, and “Telemedicine (Non-Chargeable)” is telemedicine visit, otherwise is in-person visit. |
| Sociodemographic variables |  |  |  |
| Age (years) | 3 categories | < 40 years  40-64 years  ≥65 years | Age is defined as the integer of (date – birth date)/365.25. |
| Sex | Indicator | Male/Female | Based on records in the sex column. |
| Race/Ethnicity | 6 categories | NHW  NHB  Hispanic  AAPI  Multiple  Other/unknown | Based on records in the race and ethnicity columns. |
| Primary insurance plan | 4 categories | Commercial  Medicaid  Medicare  Self-Pay/Other | Based on the PlanFCName column. “Commercial” includes “Managed Care”, “Commercial”, “Blue Cross”, and “Blue Shield”; “Medicaid” includes “Medicaid”, “Medicaid (MA)”, and “Managed Medicaid”; “Medicare” includes “Medicare” and “Managed Medicare”; all others are categorized as “Self-Pay/Other”. For encounters with multiple insurance records on the same visit date, a hierarchy was applied to assign a single primary category: Medicare -> Managed Medicare -> Medicaid -> Medicaid (MA) -> Managed Medicaid -> Commercial -> Managed Care -> Blue Cross -> Blue Shield -> Self-Pay/Other. |
| Patient portal user | Indicator | Yes/No | Based on the MyPennMedicine_Activated column. |
| Marital Status | Indicator | Yes/No | Based on the Marital_status column. |
| Encounter category | 3 categories | Return patient visit  New patient visit  Other/Unkown | Based on the enc_cat column. |
| Median household income | 3 categories | <$50,000  $50,000 to $100,000  ≥$100,000 | Based on patients’ zip code and calculated using R package tidycensus. |
| Charlson Comorbidity Index score | 2 categories | <3  ≥3 | Based on diagnosis domain. |
| Distance from home to place of service | 3 categories | <5 miles  5 to 15 miles  ≥15 miles | Based on patients’ and place of service’s zip code and calculated using R package zipcodeR. |
| Provider specialty | 3 categories | Primary care  Specialty care  Unknown | Based on eTable 2. “Primary care” includes “Family Medicine”, “Internal Medicine”, “Pediatrics”; all others are categorized as “Specialty care”. |
| Other variables |  |  |  |
| Health conditions | 7 categories | Diabetes  MBD  Sleep disorder  Heart failure  COPD  CAD  GI | Codeset available at Github: <https://github.com/Penncil/Telemedicine> |

# eTable 4. Patient-level baseline characteristics

|  | Telemedicine only (N=13,323) | In-person only (N=1,633,134) | Mixed use  (N=601,884) | Overall  (N=2,248,341) |
| --- | --- | --- | --- | --- |
| Age Range, no. (%) |  |  |  |  |
| < 40 years | 6502 (48.8) | 579623 (35.5) | 189668 (31.5) | 775793 (34.5) |
| 40-64 years | 4717 (35.4) | 600792 (36.8) | 231331 (38.4) | 836840 (37.2) |
| ≥65 years | 2104 (15.8) | 452719 (27.7) | 180885 (30.1) | 635708 (28.3) |
| Sex, no. (%) |  |  |  |  |
| Female | 7981 (59.9) | 916095 (56.1) | 377794 (62.8) | 1301870 (57.9) |
| Male | 5342 (40.1) | 717039 (43.9) | 224090 (37.2) | 946471 (42.1) |
| Race/Ethnicity, no. (%) | | | | |
| Non-Hispanic White | 6662 (50.0) | 892989 (54.7) | 379322 (63.0) | 1278973 (56.9) |
| Non-Hispanic Black | 1540 (11.6) | 254425 (15.6) | 117376 (19.5) | 373341 (16.6) |
| Hispanic | 657 (4.9) | 94393 (5.8) | 30034 (5.0) | 125084 (5.6) |
| Asian | 583 (4.4) | 94011 (5.8) | 28222 (4.7) | 122816 (5.5) |
| Other/Unknown | 3881 (29.1) | 297316 (18.2) | 46930 (7.8) | 348127 (15.5) |
| Insurance Plan, no. (%) | | | | |
| Commercial | 5534 (41.5) | 681008 (41.7) | 312968 (52.0) | 999510 (44.5) |
| Medicaid | 994 (7.5) | 131779 (8.1) | 65015 (10.8) | 197788 (8.8) |
| Medicare | 1292 (9.7) | 304457 (18.6) | 155630 (25.9) | 461379 (20.5) |
| Self-Pay/Other | 5503 (41.3) | 515890 (31.6) | 68271 (11.3) | 589664 (26.2) |
| MyPennMedicine User, no. (%) | | | | |
| No | 4750 (35.7) | 769660 (47.1) | 83579 (13.9) | 857989 (38.2) |
| Yes | 8573 (64.3) | 863474 (52.9) | 518305 (86.1) | 1390352 (61.8) |
| Marital Status, no. (%) | | | | |
| Married | 5562 (41.7) | 724320 (44.4) | 310920 (51.7) | 1040802 (46.3) |
| Unmarried | 7761 (58.3) | 908814 (55.6) | 290964 (48.3) | 1207539 (53.7) |
| Median household income, no. (%) | | | | |
| <$50,000 | 1863 (14.0) | 250928 (15.4) | 103375 (17.2) | 356166 (15.8) |
| $50,000 to $100,000 | 8073 (60.6) | 804906 (49.3) | 295929 (49.2) | 1108908 (49.3) |
| ≥$100,000 | 3387 (25.4) | 577300 (35.3) | 202580 (33.7) | 783267 (34.8) |
| Charlson Comorbidity Index score, no. (%) | | | | |
| <3 | 12822 (96.2) | 1411138 (86.4) | 398666 (66.2) | 1822626 (81.1) |
| ≥3 | 501 (3.8) | 221996 (13.6) | 203218 (33.8) | 425715 (18.9) |

# eTable 5. Odds ratios for diabetes visits

| Characteristics | Adjusted Odds Ratio (95% CI) |
| --- | --- |
| Visit Year |  |
| 2020 | 6.13 (5.90-6.38) |
| 2021 | 3.75 (3.59-3.92) |
| 2022 | 1.40 (1.34-1.46) |
| 2023 | 1.09 (1.04-1.13) |
| 2024 | 1.00 [Reference] |
| Hospital |  |
| HUP | 1.00 [Reference] |
| CCH | 0.20 (0.18-0.21) |
| PMC | 0.51 (0.49-0.53) |
| PAH | 0.82 (0.79-0.86) |
| PPMC | 0.86 (0.83-0.89) |
| Age Range |  |
| < 40 years | 1.00 [Reference] |
| 40-64 years | 0.68 (0.66-0.71) |
| ≥65 years | 0.47 (0.44-0.49) |
| Sex |  |
| Female | 1.00 [Reference] |
| Male | 0.79 (0.77-0.81) |
| Race/Ethnicity |  |
| Non-Hispanic White | 1.00 [Reference] |
| Asian | 0.94 (0.89-0.99) |
| Hispanic | 0.88 (0.82-0.93) |
| Non-Hispanic Black | 0.92 (0.89-0.96) |
| Other/Unknown | 0.90 (0.85-0.94) |
| Insurance Plan |  |
| Commercial | 1.00 [Reference] |
| Medicaid | 1.17 (1.12-1.22) |
| Medicare | 1.07 (1.03-1.11) |
| Self-Pay/Other | 0.87 (0.82-0.92) |
| MyPennMedicine User |  |
| No | 1.00 [Reference] |
| Yes | 1.66 (1.60-1.73) |
| Marital Status |  |
| Married | 1.00 [Reference] |
| Unmarried | 0.96 (0.94-0.99) |
| Median household income |  |
| <$50,000 | 1.12 (1.08-1.16) |
| $50,000 to $100,000 | 1.00 [Reference] |
| ≥$100,000 | 1.08 (1.04-1.11) |
| Charlson Comorbidity Index score |  |
| <3 | 1.00 [Reference] |
| ≥3 | 0.80 (0.77-0.82) |
| Encounter Category |  |
| Return Patient Visit | 1.00 [Reference] |
| New Patient Visit | 0.39 (0.37-0.41) |
| Other/Unknown | - |
| Distance from Home to Place of Service |  |
| < 5 miles | 1.00 [Reference] |
| 5 to 15 miles | 1.34 (1.30-1.38) |
| ≥15 miles | 1.70 (1.64-1.76) |
| Provider Specialty |  |
| Primary Care | 0.93 (0.89-0.96) |
| Specialty Care | 1.00 [Reference] |
| Unknown | 0.25 (0.24-0.26) |

# eTable 6. Odds ratios for MBD visits

| Characteristics | Adjusted Odds Ratio (95% CI) |
| --- | --- |
| Visit Year |  |
| 2020 | 1.83 (1.79-1.86) |
| 2021 | 2.09 (2.04-2.13) |
| 2022 | 1.40 (1.37-1.42) |
| 2023 | 1.09 (1.07-1.11) |
| 2024 | 1.00 [Reference] |
| Hospital |  |
| HUP | 1.00 [Reference] |
| CCH | 0.12 (0.12-0.13) |
| PMC | 0.05 (0.05-0.05) |
| PAH | 0.05 (0.05-0.05) |
| PPMC | 0.19 (0.18-0.19) |
| Age Range |  |
| < 40 years | 1.00 [Reference] |
| 40-64 years | 0.87 (0.86-0.88) |
| ≥65 years | 0.70 (0.68-0.72) |
| Sex |  |
| Female | 1.00 [Reference] |
| Male | 0.90 (0.89-0.91) |
| Race/Ethnicity |  |
| Non-Hispanic White | 1.00 [Reference] |
| Asian | 1.04 (1.01-1.07) |
| Hispanic | 0.97 (0.95-1.00) |
| Non-Hispanic Black | 0.85 (0.84-0.87) |
| Other/Unknown | 1.14 (1.11-1.16) |
| Insurance Plan |  |
| Commercial | 1.00 [Reference] |
| Medicaid | 0.64 (0.63-0.65) |
| Medicare | 0.75 (0.73-0.77) |
| Self-Pay/Other | 0.81 (0.80-0.83) |
| MyPennMedicine User |  |
| No | 1.00 [Reference] |
| Yes | 3.17 (3.10-3.23) |
| Marital Status |  |
| Married | 1.00 [Reference] |
| Unmarried | 0.91 (0.89-0.92) |
| Median household income |  |
| <$50,000 | 1.07 (1.06-1.09) |
| $50,000 to $100,000 | 1.00 [Reference] |
| ≥$100,000 | 0.87 (0.86-0.89) |
| Charlson Comorbidity Index score |  |
| <3 | 1.00 [Reference] |
| ≥3 | 0.79 (0.78-0.80) |
| Encounter Category |  |
| Return Patient Visit | 1.00 [Reference] |
| New Patient Visit | 0.42 (0.41-0.43) |
| Other/Unknown | - |
| Distance from Home to Place of Service |  |
| < 5 miles | 1.00 [Reference] |
| 5 to 15 miles | 0.96 (0.95-0.98) |
| ≥15 miles | 1.40 (1.37-1.42) |
| Provider Specialty |  |
| Primary Care | 0.58 (0.57-0.60) |
| Specialty Care | 1.00 [Reference] |
| Unknown | 0.05 (0.05-0.05) |

# eTable 7. Odds ratios for sleep awake disorder visits

| Characteristics | Adjusted Odds Ratio (95% CI) |
| --- | --- |
| Visit Year |  |
| 2020 | 2.47 (2.39-2.57) |
| 2021 | 2.32 (2.22-2.41) |
| 2022 | 1.18 (1.14-1.22) |
| 2023 | 0.96 (0.93-0.99) |
| 2024 | 1.00 [Reference] |
| Hospital |  |
| HUP | 1.00 [Reference] |
| CCH | 0.15 (0.14-0.16) |
| PMC | 0.19 (0.18-0.20) |
| PAH | 0.56 (0.54-0.57) |
| PPMC | 0.42 (0.40-0.44) |
| Age Range |  |
| < 40 years | 1.00 [Reference] |
| 40-64 years | 0.66 (0.64-0.68) |
| ≥65 years | 0.54 (0.52-0.57) |
| Sex |  |
| Female | 1.00 [Reference] |
| Male | 0.76 (0.74-0.78) |
| Race/Ethnicity |  |
| Non-Hispanic White | 1.00 [Reference] |
| Asian | 1.13 (1.06-1.20) |
| Hispanic | 0.99 (0.94-1.05) |
| Non-Hispanic Black | 1.07 (1.04-1.11) |
| Other/Unknown | 0.98 (0.94-1.03) |
| Insurance Plan |  |
| Commercial | 1.00 [Reference] |
| Medicaid | 1.05 (1.01-1.09) |
| Medicare | 1.08 (1.04-1.12) |
| Self-Pay/Other | 0.84 (0.80-0.87) |
| MyPennMedicine User |  |
| No | 1.00 [Reference] |
| Yes | 2.50 (2.40-2.61) |
| Marital Status |  |
| Married | 1.00 [Reference] |
| Unmarried | 1.04 (1.01-1.06) |
| Median household income |  |
| <$50,000 | 1.34 (1.29-1.39) |
| $50,000 to $100,000 | 1.00 [Reference] |
| ≥$100,000 | 1.06 (1.03-1.08) |
| Charlson Comorbidity Index score |  |
| <3 | 1.00 [Reference] |
| ≥3 | 0.77 (0.75-0.80) |
| Encounter Category |  |
| Return Patient Visit | 1.00 [Reference] |
| New Patient Visit | 0.45 (0.43-0.46) |
| Other/Unknown | - |
| Distance from Home to Place of Service |  |
| < 5 miles | 1.00 [Reference] |
| 5 to 15 miles | 1.00 (0.98-1.03) |
| ≥15 miles | 1.47 (1.43-1.52) |
| Provider Specialty |  |
| Primary Care | 1.20 (1.15-1.25) |
| Specialty Care | 1.00 [Reference] |
| Unknown | - |

# eTable 8. Odds ratios for heart failure visits

| Characteristics | Adjusted Odds Ratio (95% CI) |
| --- | --- |
| Visit Year |  |
| 2020 | 9.06 (8.04-10.22) |
| 2021 | 3.24 (2.81-3.73) |
| 2022 | 1.29 (1.13-1.48) |
| 2023 | 1.19 (1.04-1.36) |
| 2024 | 1.00 [Reference] |
| Hospital |  |
| HUP | 1.00 [Reference] |
| CCH | 0.26 (0.19-0.35) |
| PMC | 0.42 (0.34-0.50) |
| PAH | 0.98 (0.87-1.10) |
| PPMC | 0.68 (0.63-0.74) |
| Age Range |  |
| < 40 years | 1.00 [Reference] |
| 40-64 years | 0.89 (0.76-1.03) |
| ≥65 years | 0.85 (0.72-1.00) |
| Sex |  |
| Female | 1.00 [Reference] |
| Male | 0.70 (0.65-0.75) |
| Race/Ethnicity |  |
| Non-Hispanic White | 1.00 [Reference] |
| Asian | 1.09 (0.85-1.40) |
| Hispanic | 0.89 (0.71-1.11) |
| Non-Hispanic Black | 1.00 (0.91-1.10) |
| Other/Unknown | 0.86 (0.72-1.01) |
| Insurance Plan |  |
| Commercial | 1.00 [Reference] |
| Medicaid | 1.23 (1.08-1.40) |
| Medicare | 1.10 (0.99-1.22) |
| Self-Pay/Other | 1.07 (0.91-1.27) |
| MyPennMedicine User |  |
| No | 1.00 [Reference] |
| Yes | 2.17 (1.07-1.28) |
| Marital Status |  |
| Married | 1.00 [Reference] |
| Unmarried | 0.97 (0.90-1.04) |
| Median household income |  |
| <$50,000 | 1.11 (1.00-1.23) |
| $50,000 to $100,000 | 1.00 [Reference] |
| ≥$100,000 | 1.13 (1.04-1.24) |
| Charlson Comorbidity Index score |  |
| <3 | 1.00 [Reference] |
| ≥3 | 0.71 (0.65-0.78) |
| Encounter Category |  |
| Return Patient Visit | 1.00 [Reference] |
| New Patient Visit | 0.40 (0.35-0.45) |
| Other/Unknown | - |
| Distance from Home to Place of Service |  |
| < 5 miles | 1.00 [Reference] |
| 5 to 15 miles | 1.11 (1.01-1.22) |
| ≥15 miles | 1.57 (1.42-1.73) |
| Provider Specialty |  |
| Primary Care | 2.65 (2.31-3.04) |
| Specialty Care | 1.00 [Reference] |
| Unknown | 0.27 (0.23-0.32) |

# eTable 9. Odds ratios for COPD visits

| Characteristics | Adjusted Odds Ratio (95% CI) |
| --- | --- |
| Visit Year |  |
| 2020 | 8.88 (7.69-10.25) |
| 2021 | 5.00 (4.25-5.88) |
| 2022 | 1.63 (1.40-1.90) |
| 2023 | 1.09 (0.93-1.28) |
| 2024 | 1.00 [Reference] |
| Hospital |  |
| HUP | 1.00 [Reference] |
| CCH | 0.44 (0.33-0.58) |
| PMC | 0.68 (0.58-0.80) |
| PAH | 1.29 (1.15-1.46) |
| PPMC | 1.08 (0.97-1.21) |
| Age Range |  |
| < 40 years | 1.00 [Reference] |
| 40-64 years | 1.60 (0.77-3.30) |
| ≥65 years | 1.26 (0.60-2.61) |
| Sex |  |
| Female | 1.00 [Reference] |
| Male | 0.77 (0.71-0.84) |
| Race/Ethnicity |  |
| Non-Hispanic White | 1.00 [Reference] |
| Asian | 0.93 (0.63-1.38) |
| Hispanic | 1.52 (1.13-2.04) |
| Non-Hispanic Black | 1.18 (1.05-1.33) |
| Other/Unknown | 0.87 (0.69-1.08) |
| Insurance Plan |  |
| Commercial | 1.00 [Reference] |
| Medicaid | 1.43 (1.21-1.69) |
| Medicare | 1.08 (0.94-1.25) |
| Self-Pay/Other | 1.32 (1.07-1.62) |
| MyPennMedicine User |  |
| No | 1.00 [Reference] |
| Yes | 1.35 (1.22-1.49) |
| Marital Status |  |
| Married | 1.00 [Reference] |
| Unmarried | 1.12 (1.02-1.22) |
| Median household income |  |
| <$50,000 | 1.16 (1.02-1.31) |
| $50,000 to $100,000 | 1.00 [Reference] |
| ≥$100,000 | 1.06 (0.95-1.18) |
| Charlson Comorbidity Index score |  |
| <3 | 1.00 [Reference] |
| ≥3 | 0.70 (0.63-0.77) |
| Encounter Category |  |
| Return Patient Visit | 1.00 [Reference] |
| New Patient Visit | 0.29 (0.25-0.34) |
| Other/Unknown | - |
| Distance from Home to Place of Service |  |
| < 5 miles | 1.00 [Reference] |
| 5 to 15 miles | 0.88 (0.80-0.98) |
| ≥15 miles | 1.49 (1.31-1.68) |
| Provider Specialty |  |
| Primary Care | 1.49 (1.30-1.71) |
| Specialty Care | 1.00 [Reference] |
| Unknown | - |

# eTable 10. Odds ratios for CAD visits

| Characteristics | Adjusted Odds Ratio (95% CI) |
| --- | --- |
| Visit Year |  |
| 2020 | 11.38 (10.12-12.80) |
| 2021 | 4.31 (3.77-4.93) |
| 2022 | 1.39 (1.21-1.59) |
| 2023 | 1.04 (0.91-1.20) |
| 2024 | 1.00 [Reference] |
| Hospital |  |
| HUP | 1.00 [Reference] |
| CCH | 0.31 (0.25-0.38) |
| PMC | 0.54 (0.47-0.62) |
| PAH | 1.02 (0.93-1.12) |
| PPMC | 0.55 (0.50-0.60) |
| Age Range |  |
| < 40 years | 1.00 [Reference] |
| 40-64 years | 0.47 (0.35-0.63) |
| ≥65 years | 0.40 (0.30-0.54) |
| Sex |  |
| Female | 1.00 [Reference] |
| Male | 0.84 (0.78-0.90) |
| Race/Ethnicity |  |
| Non-Hispanic White | 1.00 [Reference] |
| Asian | 1.11 (0.94-1.32) |
| Hispanic | 0.95 (0.76-1.19) |
| Non-Hispanic Black | 1.12 (1.00-1.26) |
| Other/Unknown | 0.98 (0.84-1.14) |
| Insurance Plan |  |
| Commercial | 1.00 [Reference] |
| Medicaid | 1.02 (0.86-1.21) |
| Medicare | 0.96 (0.87-1.07) |
| Self-Pay/Other | 0.96 (0.81-1.14) |
| MyPennMedicine User |  |
| No | 1.00 [Reference] |
| Yes | 1.39 (1.27-1.53) |
| Marital Status |  |
| Married | 1.00 [Reference] |
| Unmarried | 0.98 (0.91-1.06) |
| Median household income |  |
| <$50,000 | 1.10 (0.98-1.24) |
| $50,000 to $100,000 | 1.00 [Reference] |
| ≥$100,000 | 1.04 (0.96-1.12) |
| Charlson Comorbidity Index score |  |
| <3 | 1.00 [Reference] |
| ≥3 | 0.77 (0.71-0.84) |
| Encounter Category |  |
| Return Patient Visit | 1.00 [Reference] |
| New Patient Visit | 0.31 (0.28-0.35) |
| Other/Unknown | - |
| Distance from Home to Place of Service |  |
| < 5 miles | 1.00 [Reference] |
| 5 to 15 miles | 1.15 (1.06-1.25) |
| ≥15 miles | 1.72 (1.57-1.88) |
| Provider Specialty |  |
| Primary Care | 1.78 (1.57-2.03) |
| Specialty Care | 1.00 [Reference] |
| Unknown | 0.19 (0.15-0.24) |

# eTable 11. Odds ratios for GI visits

| Characteristics | Adjusted Odds Ratio (95% CI) |
| --- | --- |
| Visit Year |  |
| 2020 | 6.47 (6.27-6.68) |
| 2021 | 4.43 (4.27-4.59) |
| 2022 | 1.20 (1.15-1.24) |
| 2023 | 0.96 (0.93-1.00) |
| 2024 | 1.00 [Reference] |
| Hospital |  |
| HUP | 1.00 [Reference] |
| CCH | 0.14 (0.14-0.15) |
| PMC | 0.77 (0.74-0.80) |
| PAH | 1.03 (1.00-1.06) |
| PPMC | 0.82 (0.80-0.85) |
| Age Range |  |
| < 40 years | 1.00 [Reference] |
| 40-64 years | 0.80 (0.78-0.82) |
| ≥65 years | 0.62 (0.60-0.65) |
| Sex |  |
| Female | 1.00 [Reference] |
| Male | 0.79 (0.78-0.81) |
| Race/Ethnicity |  |
| Non-Hispanic White | 1.00 [Reference] |
| Asian | 0.79 (0.75-0.83) |
| Hispanic | 0.96 (0.91-1.01) |
| Non-Hispanic Black | 1.05 (1.02-1.08) |
| Other/Unknown | 0.87 (0.83-0.91) |
| Insurance Plan |  |
| Commercial | 1.00 [Reference] |
| Medicaid | 1.15 (1.11-1.19) |
| Medicare | 1.11 (1.08-1.15) |
| Self-Pay/Other | 0.97 (0.93-1.01) |
| MyPennMedicine User |  |
| No | 1.00 [Reference] |
| Yes | 1.53 (1.49-1.59) |
| Marital Status |  |
| Married | 1.00 [Reference] |
| Unmarried | 1.00 (0.98-1.02) |
| Median household income |  |
| <$50,000 | 1.09 (1.06-1.13) |
| $50,000 to $100,000 | 1.00 [Reference] |
| ≥$100,000 | 1.00 (0.98-1.02) |
| Charlson Comorbidity Index score |  |
| <3 | 1.00 [Reference] |
| ≥3 | 0.87 (0.85-0.89) |
| Encounter Category |  |
| Return Patient Visit | 1.00 [Reference] |
| New Patient Visit | 0.29 (0.29-0.30) |
| Other/Unknown | - |
| Distance from Home to Place of Service |  |
| < 5 miles | 1.00 [Reference] |
| 5 to 15 miles | 1.12 (1.09-1.15) |
| ≥15 miles | 1.44 (1.40-1.48) |
| Provider Specialty |  |
| Primary Care | 1.37 (1.32-1.41) |
| Specialty Care | 1.00 [Reference] |
| Unknown | - |

# eTable 12. Baseline characteristics for different health systems

|  | HUP | CCH | PMC | PATH | PPMC |
| --- | --- | --- | --- | --- | --- |
| *Patient level* | | | | | |
| No. of patients | 1,434,416 | 321,410 | 362,692 | 566,814 | 655,190 |
| Age Range, no. (%) |  |  |  |  |  |
| < 40 years | 464688 (32.4) | 105772 (32.9) | 125075 (34.5) | 220162 (38.8) | 210692 (32.2) |
| 40-64 years | 546076 (38.1) | 125959 (39.2) | 138314 (38.1) | 211777 (37.4) | 250574 (38.2) |
| ≥65 years | 423652 (29.5) | 89679 (27.9) | 99303 (27.4) | 134875 (23.8) | 193924 (29.6) |
| Sex, no. (%) |  |  |  |  |  |
| Female | 841391 (58.7) | 188906 (58.8) | 212277 (58.5) | 353240 (62.3) | 379040 (57.9) |
| Male | 593025 (41.3) | 132504 (41.2) | 150415 (41.5) | 213574 (37.7) | 276150 (42.1) |
| Race/Ethnicity, no. (%) | | | | |  |
| Non-Hispanic White | 835215 (58.2) | 235856 (73.4) | 206036 (56.8) | 299442 (52.8) | 339057 (51.7) |
| Non-Hispanic Black | 252252 (17.6) | 24084 (7.5) | 29999 (8.3) | 149075 (26.3) | 187751 (28.7) |
| Hispanic | 56719 (4.0) | 25283 (7.9) | 28810 (7.9) | 33881 (6.0) | 29867 (4.6) |
| Asian | 60694 (4.2) | 11480 (3.6) | 42979 (11.9) | 29217 (5.2) | 30988 (4.7) |
| Other/Unknown | 229536 (16.0) | 24707 (7.7) | 54868 (15.1) | 55199 (9.7) | 67527 (10.3) |
| Insurance Plan, no. (%) | | | | |  |
| Commercial | 652787 (45.5) | 167922 (52.2) | 170600 (47.0) | 262162 (46.3) | 293732 (44.8) |
| Medicaid | 132500 (9.2) | 19953 (6.2) | 8693 (2.4) | 87258 (15.4) | 88024 (13.4) |
| Medicare | 317734 (22.2) | 66424 (20.7) | 67514 (18.6) | 115540 (20.4) | 161756 (24.7) |
| Self-Pay/Other | 331395 (23.1) | 67111 (20.9) | 115885 (32.0) | 101854 (18.0) | 111678 (17.0) |
| MyPennMedicine User, no. (%) | | | | |  |
| No | 451538 (31.5) | 109230 (34.0) | 166655 (45.9) | 136411 (24.1) | 165819 (25.3) |
| Yes | 982878 (68.5) | 212180 (66.0) | 196037 (54.1) | 430403 (75.9) | 489371 (74.7) |
| Marital Status, no. (%) | | | | |  |
| Married | 680622 (47.4) | 172760 (53.8) | 186678 (51.5) | 244392 (43.1) | 296270 (45.2) |
| Unmarried | 753794 (52.6) | 148650 (46.2) | 176014 (48.5) | 322422 (56.9) | 358920 (54.8) |
| Median household income, no. (%) | | | | |  |
| <$50,000 | 245512 (17.1) | 8459 (2.6) | 17095 (4.7) | 146673 (25.9) | 183628 (28.0) |
| $50,000 to $100,000 | 726547 (50.7) | 135622 (42.2) | 138195 (38.1) | 304837 (53.8) | 329473 (50.3) |
| ≥$100,000 | 462357 (32.2) | 177329 (55.2) | 207402 (57.2) | 115304 (20.3) | 142089 (21.7) |
| Charlson Comorbidity Index score, no. (%) | | | | |  |
| <3 | 1109196 (77.3) | 240326 (74.8) | 304418 (83.9%) | 429255 (75.7) | 469087 (71.6) |
| ≥3 | 325220 (22.7) | 81084 (25.2) | 58274 (16.1%) | 137559 (24.3) | 186103 (28.4) |
| *Encounter level* | | | | | |
| No. of visits | 32,579,604 | 4,226,757 | 6,274,330 | 6,573,163 | 7,495,880 |
| Provider Specialty, no. (%) | | | | | |
| Primary Care | 1018325 (4.7) | 742919 (17.6) | 286469 (4.6) | 4529237 (68.9) | 727333 (9.7) |
| Specialty Care | 13250860 (61.4) | 1952245 (46.2) | 3340639 (53.2) | 1850588 (28.2) | 4287326 (57.2) |
| Unknown | 7310419 (33.9) | 1531593 (36.2) | 2647222 (42.2) | 4529237 (68.9) | 2481221 (33.1) |
| Encounter Category, no. (%) | | | | | |
| Return Patient Visit | 11469560 (53.2) | 2199952 (52.0) | 3606593 (57.5) | 3610622 (54.9) | 4179677 (55.8) |
| New Patient Visit | 2291927 (10.6) | 240316 (5.7) | 341085 (5.4) | 792990 (12.1) | 857601 (11.4) |
| Other/Unknown | 7818117 (36.2) | 1786489 (42.3) | 2326652 (37.1) | 2169551 (33.0) | 2458602 (32.8) |
| Distance from Home to Place of Service, no. (%) | | | | | |
| < 5 miles | 6864570 (31.8) | 1187567 (28.1) | 1699823 (27.1) | 3363906 (51.2) | 3675927 (49.0) |
| 5 to 15 miles | 7570634 (35.1) | 2273204 (53.8) | 3139244 (50.0) | 2000170 (30.4) | 2265883 (30.2) |
| ≥15 miles | 7144400 (33.1) | 765986 (18.1) | 1435263 (22.9) | 1209087 (18.4) | 1554070 (20.7) |

# eTable 13. Odds ratios with a finer age category

| Characteristics | Adjusted Odds Ratio (95% CI) |
| --- | --- |
| Visit Year |  |
| 2019 | 1.00 [Reference] |
| 2020 | 17.87 (17.70-18.04) |
| 2021 | 11.44 (11.33-11.55) |
| 2022 | 8.12 (8.04-8.20) |
| 2023 | 6.59 (6.53-6.66) |
| 2024 | 6.23 (6.17-6.30) |
| Hospital |  |
| HUP | 1.00 [Reference] |
| CCH | 0.45 (0.44-0.45) |
| PMC | 1.18 (1.17-1.18) |
| PAH | 0.57 (0.57-0.58) |
| PPMC | 0.59 (0.58-0.59) |
| Age Range |  |
| < 40 years | 1.00 [Reference] |
| 40-64 years | 0.67 (0.67-0.67) |
| 65-74 years | 0.50 (0.50-0.51) |
| ≥75 years | 0.41 (0.41-0.42) |
| Sex |  |
| Female | 1.00 [Reference] |
| Male | 0.90 (0.90-0.91) |
| Race/Ethnicity |  |
| Non-Hispanic White | 1.00 [Reference] |
| Asian | 0.82 (0.82-0.83) |
| Hispanic | 0.94 (0.93-0.94) |
| Non-Hispanic Black | 0.88 (0.88-0.88) |
| Other/Unknown | 0.96 (0.96-0.97) |
| Insurance Plan |  |
| Commercial | 1.00 [Reference] |
| Medicaid | 0.99 (0.99-1.00) |
| Medicare | 1.10 (1.09-1.11) |
| Self-Pay/Other | 1.14 (1.14-1.15) |
| MyPennMedicine User |  |
| No | 1.00 [Reference] |
| Yes | 1.42 (1.42-1.43) |
| Marital Status |  |
| Married | 1.00 [Reference] |
| Unmarried | 1.11 (1.10-1.11) |
| Median household income |  |
| <$50,000 | 1.06 (1.05-1.06) |
| $50,000 to $100,000 | 1.00 [Reference] |
| ≥$100,000 | 0.92 (0.91-0.92) |
| Charlson Comorbidity Index score |  |
| <3 | 1.00 [Reference] |
| ≥3 | 0.88 (0.87-0.88) |
| Encounter Category |  |
| Return Patient Visit | 1.00 [Reference] |
| New Patient Visit | 0.46 (0.46-0.47) |
| Other/Unknown | 0.27 (0.27-0.27) |
| Distance from Home to Place of Service |  |
| < 5 miles | 1.00 [Reference] |
| 5 to 15 miles | 1.03 (1.03-1.04) |
| ≥15 miles | 1.41 (1.40-1.41) |
| Provider Specialty |  |
| Primary Care | 1.23 (1.22-1.23) |
| Specialty Care | 1.00 [Reference] |
| Unknown | 0.06 (0.06-0.06) |

# eTable 14. Odds ratios with the interaction term of MBD and race/ethnicity

| Characteristics | Adjusted Odds Ratio (95% CI) |
| --- | --- |
| Visit Year |  |
| 2019 | 1.00 [Reference] |
| 2020 | 18.59 (18.41-18.77) |
| 2021 | 12.35 (12.23-12.47) |
| 2022 | 8.30 (8.22-8.38) |
| 2023 | 6.74 (6.67-6.80) |
| 2024 | 6.38 (6.34-6.44) |
| Hospital |  |
| HUP | 1.00 [Reference] |
| CCH | 0.45 (0.45-0.46) |
| PMC | 1.10 (1.09-1.10) |
| PAH | 0.56 (0.56-0.56) |
| PPMC | 0.61 (0.61-0.61) |
| Age Range |  |
| < 40 years | 1.00 [Reference] |
| 40-64 years | 0.71 (0.71-0.72) |
| ≥65 years | 0.53 (0.53-0.54) |
| Sex |  |
| Female | 1.00 [Reference] |
| Male | 0.89 (0.88-0.89) |
| Race/Ethnicity |  |
| Non-Hispanic White | 1.00 [Reference] |
| Asian | 0.84 (0.84-0.85) |
| Hispanic | 1.02 (1.01-1.03) |
| Non-Hispanic Black | 0.98 (0.97-0.98) |
| Other/Unknown | 0.97 (0.97-0.98) |
| Insurance Plan |  |
| Commercial | 1.00 [Reference] |
| Medicaid | 0.93 (0.92-0.93) |
| Medicare | 1.04 (1.04-1.05) |
| Self-Pay/Other | 1.09 (1.08-1.09) |
| MyPennMedicine User |  |
| No | 1.00 [Reference] |
| Yes | 1.51 (1.51-1.52) |
| Marital Status |  |
| Married | 1.00 [Reference] |
| Unmarried | 1.04 (1.04-1.05) |
| Median household income |  |
| <$50,000 | 1.05 (1.04-1.05) |
| $50,000 to $100,000 | 1.00 [Reference] |
| ≥$100,000 | 0.93 (0.93-0.93) |
| Charlson Comorbidity Index score |  |
| <3 | 1.00 [Reference] |
| ≥3 | 0.90 (0.90-0.90) |
| Encounter Category |  |
| Return Patient Visit | 1.00 [Reference] |
| New Patient Visit | 0.50 (0.49-0.50) |
| Other/Unknown | 0.29 (0.29-0.29) |
| Distance from Home to Place of Service |  |
| < 5 miles | 1.00 [Reference] |
| 5 to 15 miles | 1.07 (1.06-1.07) |
| ≥15 miles | 1.48 (1.47-1.49) |
| Provider Specialty |  |
| Primary Care | 1.23 (1.23-1.24) |
| Specialty Care | 1.00 [Reference] |
| Unknown | 0.07 (0.07-0.07) |
| MBD × Race/Ethnicity |  |
| MBD (1)×Asian | 0.90 (0.87-0.91) |
| MBD (1)×Hispanic | 0.64 (0.62-0.65) |
| MBD (1)×Non-Hispanic Black | 0.61 (0.60-0.62) |
| MBD (1)×Other/Unknown | 0.95 (0.94-0.97) |

# eTable 15. Odds ratios with the interaction term of visit year and insurance within MBD visits

| Characteristics | Adjusted Odds Ratio (95% CI) |
| --- | --- |
| Visit Year |  |
| 2020 | 1.87 (1.81-1.92) |
| 2021 | 2.32 (2.24-2.39) |
| 2022 | 1.48 (1.44-1.52) |
| 2023 | 1.10 (1.07-1.13) |
| 2024 | 1.00 [Reference] |
| Hospital |  |
| HUP | 1.00 [Reference] |
| CCH | 0.12 (0.12-0.13) |
| PMC | 0.05 (0.05-0.05) |
| PAH | 0.05 (0.05-0.05) |
| PPMC | 0.19 (0.18-0.19) |
| Age Range |  |
| < 40 years | 1.00 [Reference] |
| 40-64 years | 0.87 (0.85-0.88) |
| ≥65 years | 0.71 (0.69-0.73) |
| Sex |  |
| Female | 1.00 [Reference] |
| Male | 0.90 (0.89-0.91) |
| Race/Ethnicity |  |
| Non-Hispanic White | 1.00 [Reference] |
| Asian | 1.04 (1.01-1.07) |
| Hispanic | 0.98 (0.95-1.00) |
| Non-Hispanic Black | 0.85 (0.84-0.87) |
| Other/Unknown | 1.13 (1.11-1.16) |
| Insurance Plan |  |
| Commercial | 1.00 [Reference] |
| Medicaid | 0.77 (0.74-0.80) |
| Medicare | 0.81 (0.78-0.84) |
| Self-Pay/Other | 0.79 (0.77-0.82) |
| MyPennMedicine User |  |
| No | 1.00 [Reference] |
| Yes | 3.19 (3.12-3.25) |
| Marital Status |  |
| Married | 1.00 [Reference] |
| Unmarried | 0.90 (0.89-0.92) |
| Median household income |  |
| <$50,000 | 1.08 (1.06-1.10) |
| $50,000 to $100,000 | 1.00 [Reference] |
| ≥$100,000 | 0.87 (0.86-0.89) |
| Charlson Comorbidity Index score |  |
| <3 | 1.00 [Reference] |
| ≥3 | 0.79 (0.78-0.81) |
| Encounter Category |  |
| Return Patient Visit | 1.00 [Reference] |
| New Patient Visit | 0.42 (0.41-0.43) |
| Other/Unknown | - |
| Distance from Home to Place of Service |  |
| < 5 miles | 1.00 [Reference] |
| 5 to 15 miles | 0.96 (0.95-0.98) |
| ≥15 miles | 1.40 (1.37-1.42) |
| Provider Specialty |  |
| Primary Care | 0.58 (0.57-0.60) |
| Specialty Care | 1.00 [Reference] |
| Unknown | 0.05 (0.05-0.05) |
| Visit Year × Insurance Plan |  |
| 2020 × Medicaid | 0.75 (0.71-0.78) |
| 2020 × Medicare | 1.01 (0.95-1.07) |
| 2020 × Self-Pay/Other | 1.56 (1.45-1.68) |
| 2021 × Medicaid | 0.61 (0.58-0.64) |
| 2021 × Medicare | 0.82 (0.77-0.87) |
| 2021 × Self-Pay/Other | 2.54 (2.31-2.80) |
| 2022 × Medicaid | 0.84 (0.80-0.88) |
| 2022 × Medicare | 0.86 (0.82-0.90) |
| 2022 × Self-Pay/Other | 0.93 (0.88-0.97) |
| 2023 × Medicaid | 1.02 (0.97-1.07) |
| 2023 × Medicare | 0.93 (0.88-0.98) |
| 2023 × Self-Pay/Other | 0.99 (0.95-1.04) |

# eTable 16. Crude odds ratios

| Characteristics | Crude Odds Ratio (95% CI) |
| --- | --- |
| Visit Year |  |
| 2019 | 1.00 [Reference] |
| 2020 | 16.51 (16.35-16.66) |
| 2021 | 10.14 (10.04-10.23) |
| 2022 | 7.77 (7.70-7.85) |
| 2023 | 6.53 (6.47-6.59) |
| 2024 | 6.35 (6.28-6.41) |
| Hospital |  |
| HUP | 1.00 [Reference] |
| CCH | 0.39 (0.39-0.39) |
| PMC | 0.76 (0.75-0.76) |
| PAH | 0.68 (0.68-0.68) |
| PPMC | 0.61 (0.60-0.61) |
| Age Range |  |
| < 40 years | 1.00 [Reference] |
| 40-64 years | 0.66 (0.65-0.66) |
| ≥65 years | 0.44 (0.44-0.44) |
| Sex |  |
| Female | 1.00 [Reference] |
| Male | 0.84 (0.84-0.84) |
| Race/Ethnicity |  |
| Non-Hispanic White | 1.00 [Reference] |
| Asian | 0.88 (0.88-0.89) |
| Hispanic | 1.06 (1.06-1.07) |
| Non-Hispanic Black | 0.91 (0.91-0.92) |
| Other/Unknown | 1.05 (1.05-1.06) |
| Insurance Plan |  |
| Commercial | 1.00 [Reference] |
| Medicaid | 0.93 (0.93-0.93) |
| Medicare | 0.63 (0.62-0.63) |
| Self-Pay/Other | 0.79 (0.79-0.80) |
| MyPennMedicine User |  |
| No | 1.00 [Reference] |
| Yes | 1.80 (1.79-1.81) |
| Marital Status |  |
| Married | 1.00 [Reference] |
| Unmarried | 1.16 (1.16-1.16) |
| Median household income |  |
| <$50,000 | 0.95 (0.95-0.95) |
| $50,000 to $100,000 | 1.00 [Reference] |
| ≥$100,000 | 0.90 (0.90-0.90) |
| Charlson Comorbidity Index score |  |
| <3 | 1.00 [Reference] |
| ≥3 | 0.71 (0.71-0.71) |
| Encounter Category |  |
| Return Patient Visit | 1.00 [Reference] |
| New Patient Visit | 0.64 (0.63-0.64) |
| Other/Unknown | 0.18 (0.18-0.19) |
| Distance from Home to Place of Service |  |
| < 5 miles | 1.00 [Reference] |
| 5 to 15 miles | 0.97 (0.97-0.98) |
| ≥15 miles | 1.32 (1.31-1.32) |
| Provider Specialty |  |
| Primary Care | 1.19 (1.18-1.19) |
| Specialty Care | 1.00 [Reference] |
| Unknown | 0.06 (0.06-0.06) |

# eFigure 1. Temporal patterns for diabetes visits


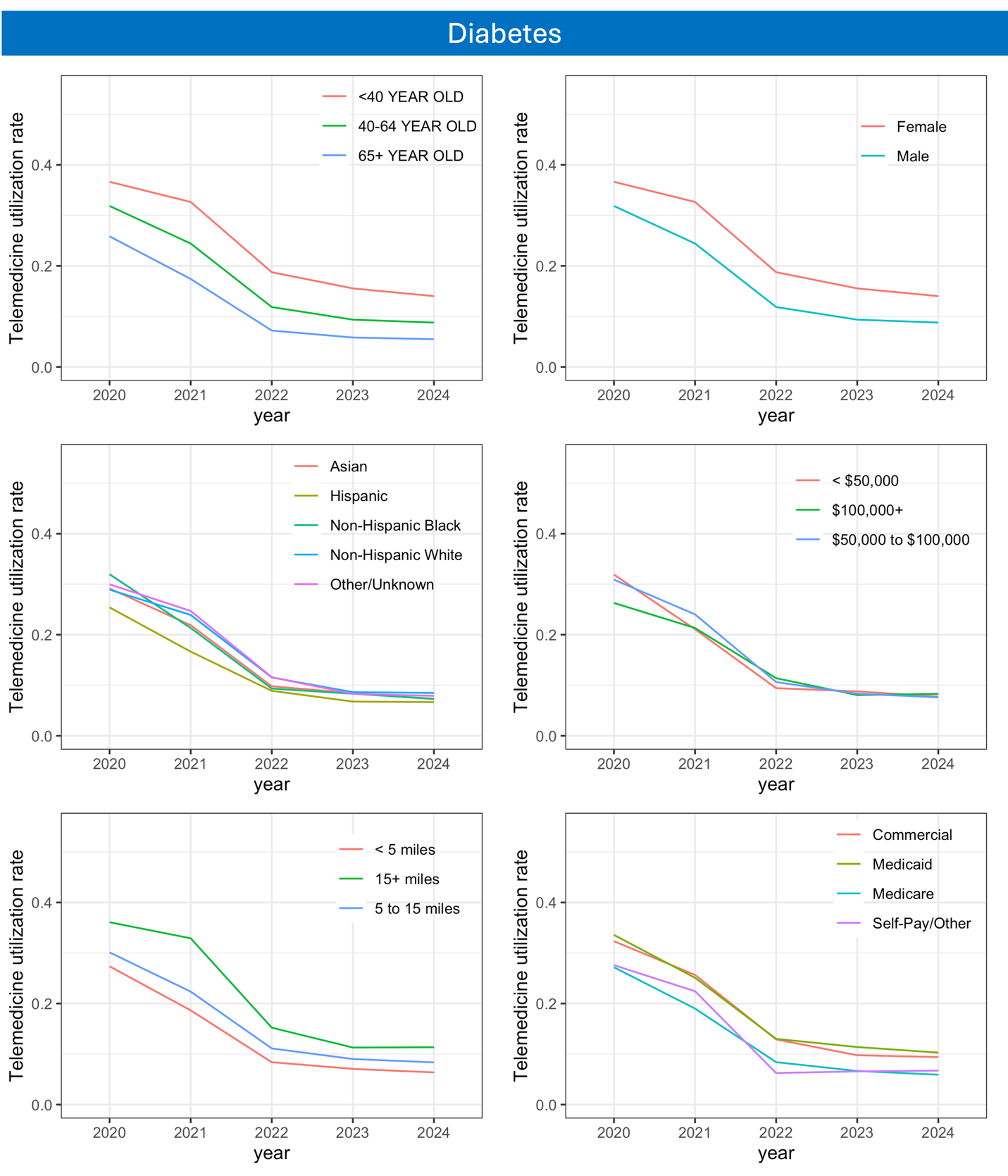


# eFigure 2. Temporal patterns for MBD visits


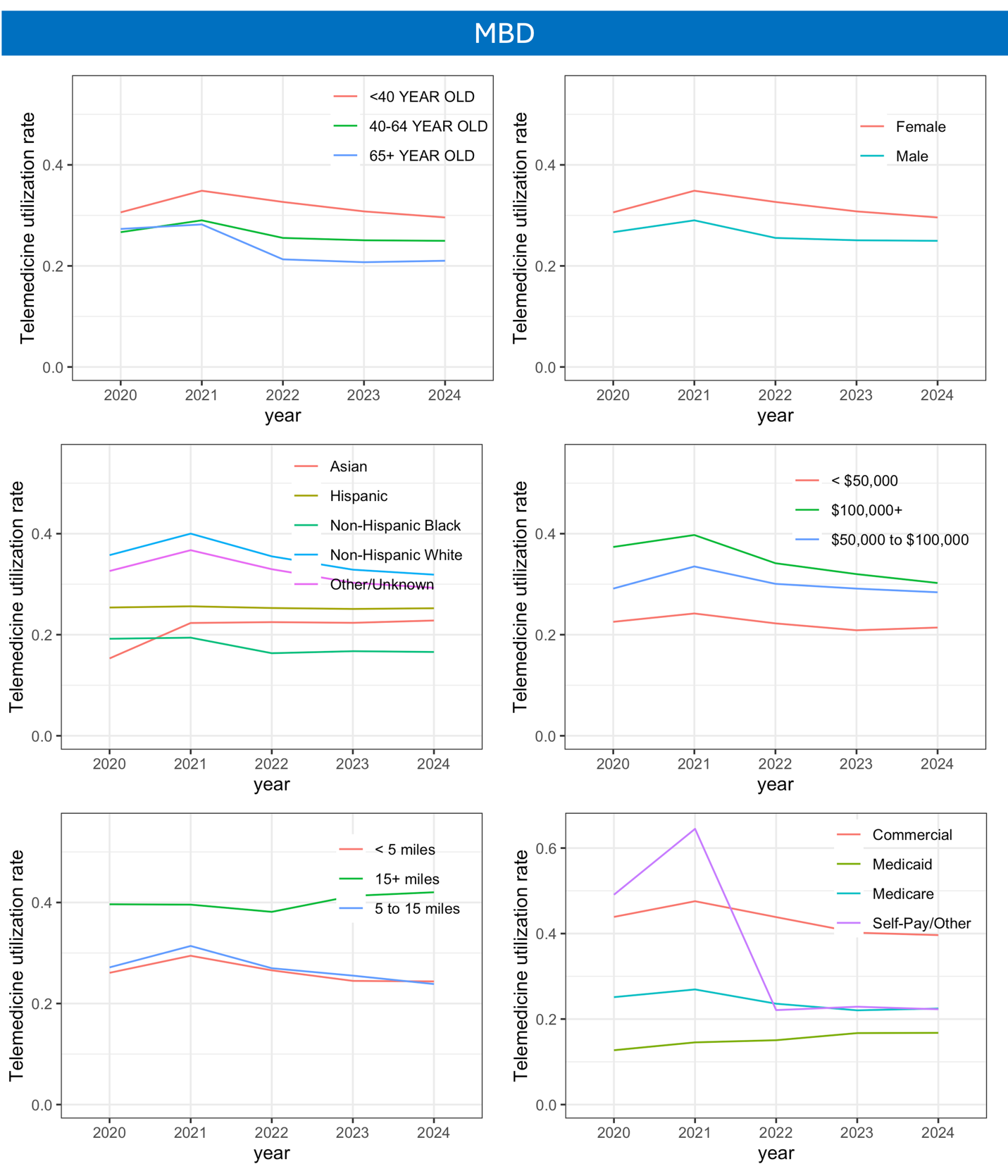


# eFigure 3. Temporal patterns for sleep awake disorder visits


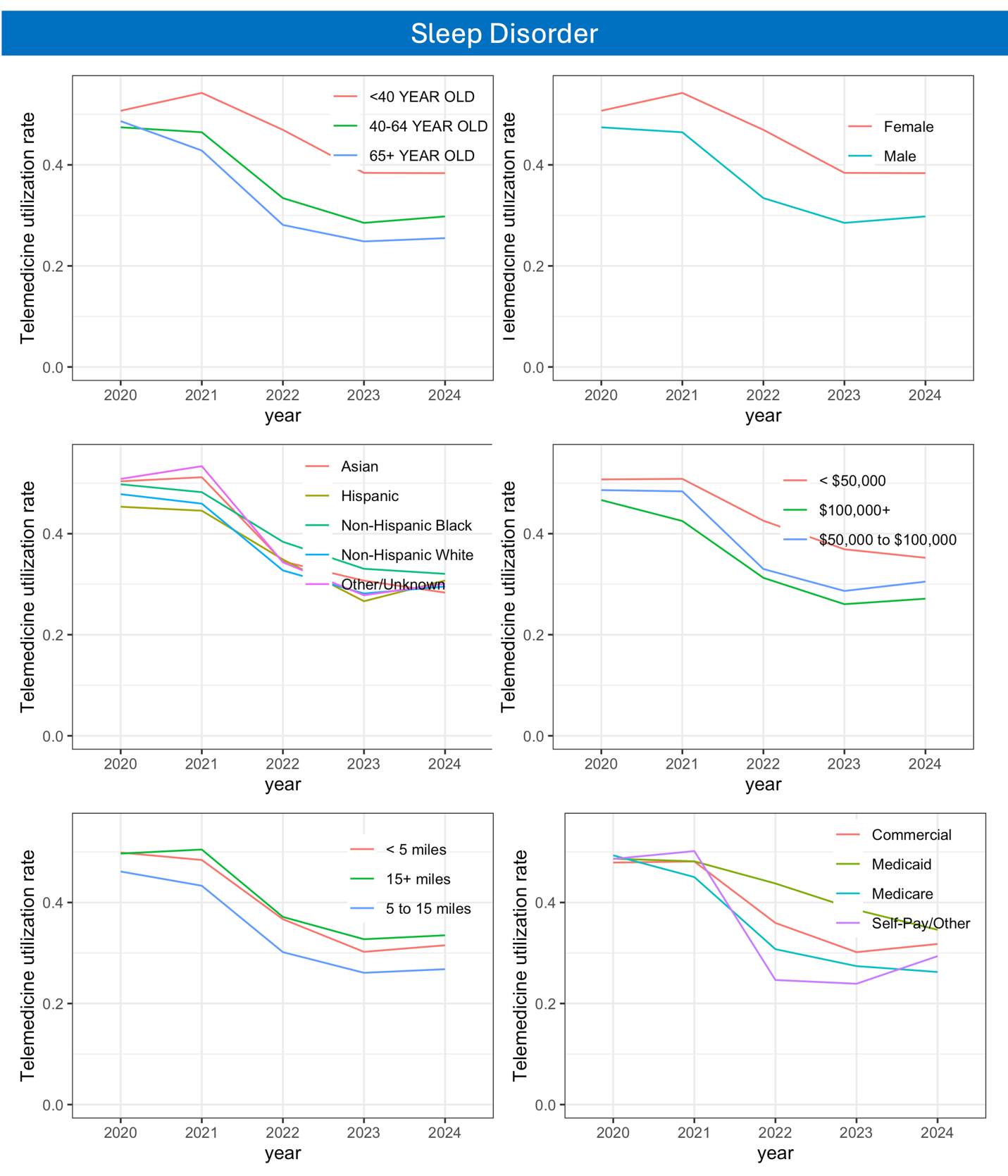


# eFigure 4. Temporal patterns for heart failure visits


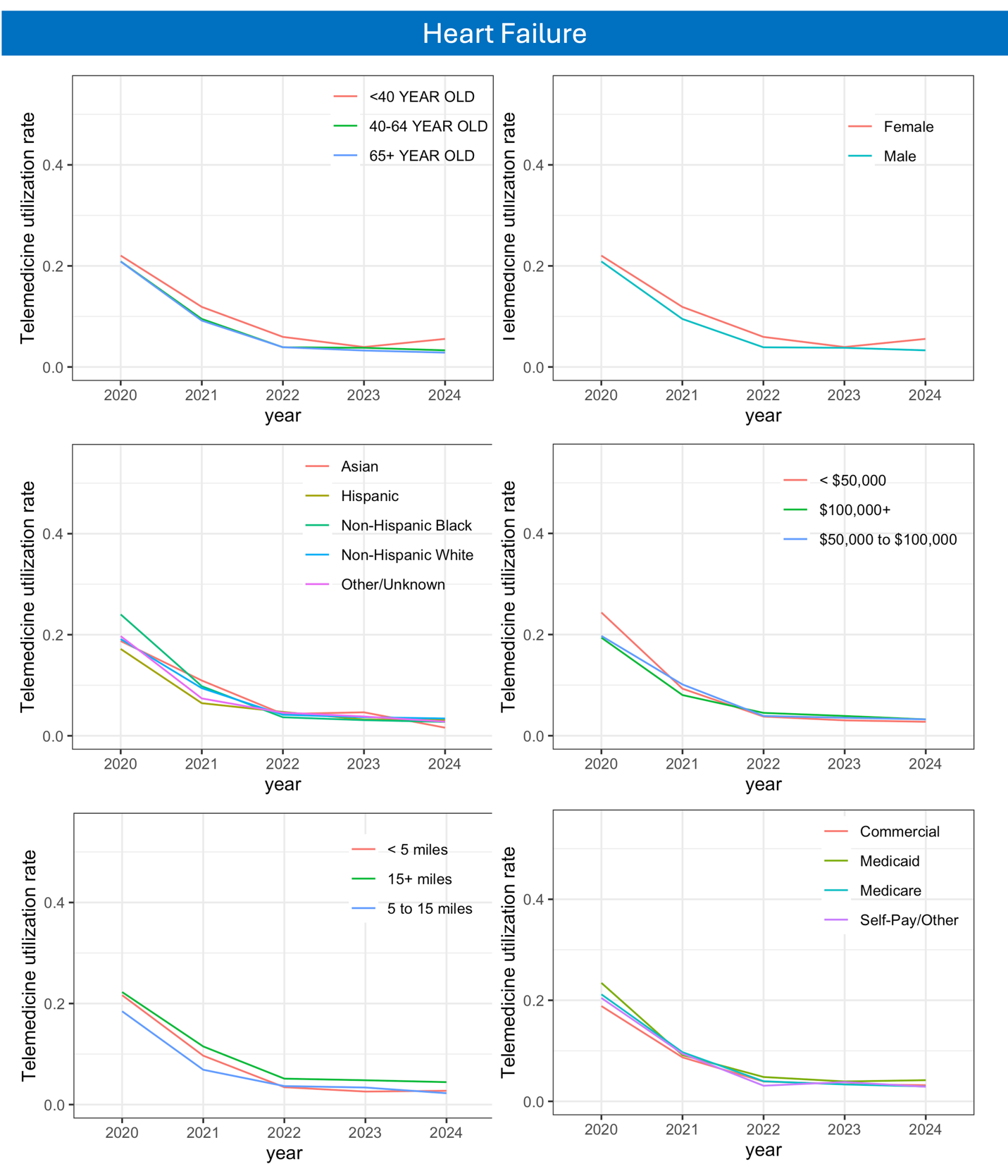


# eFigure 5. Temporal patterns for COPD visits


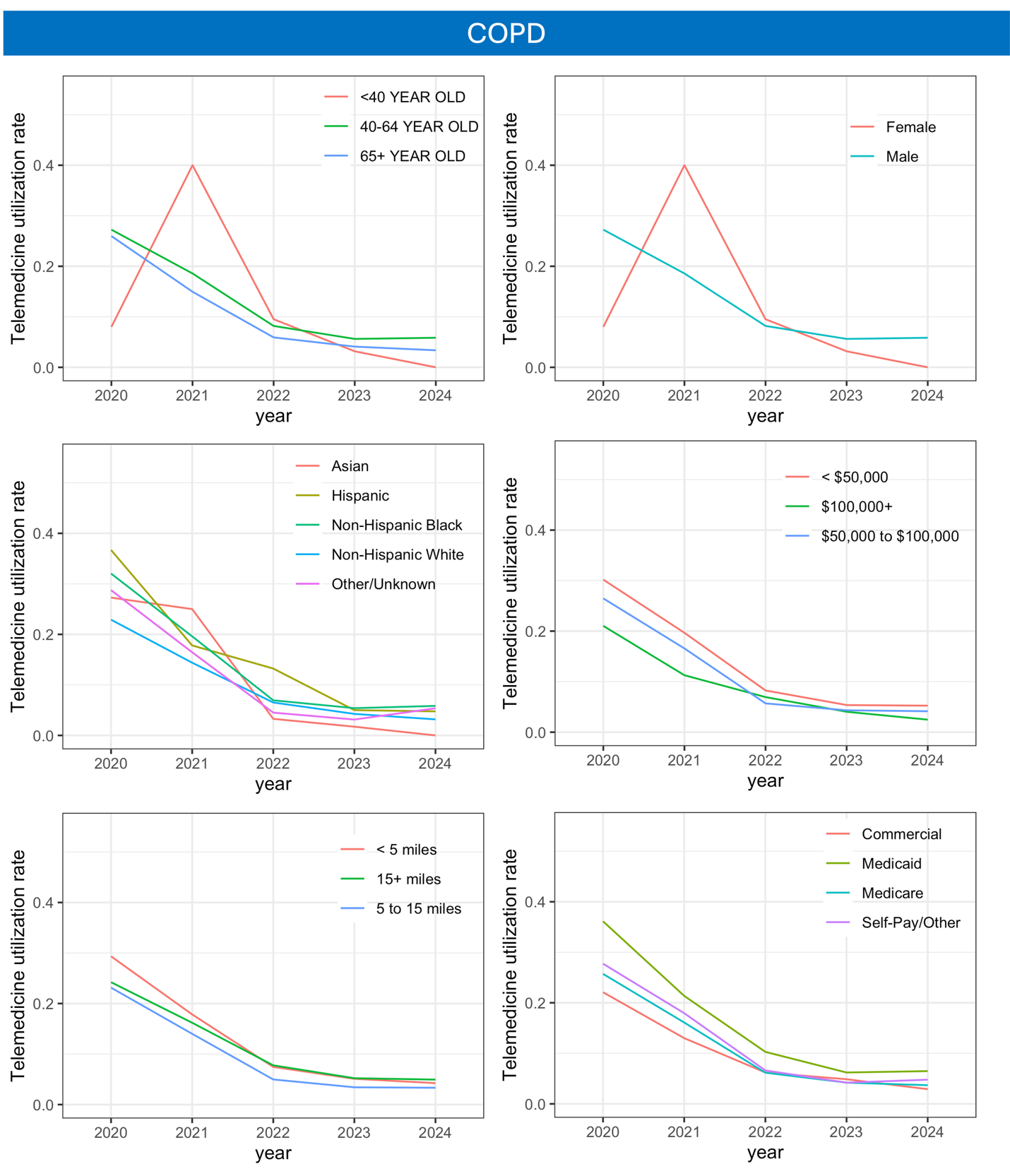


# eFigure 6. Temporal patterns for CAD visits


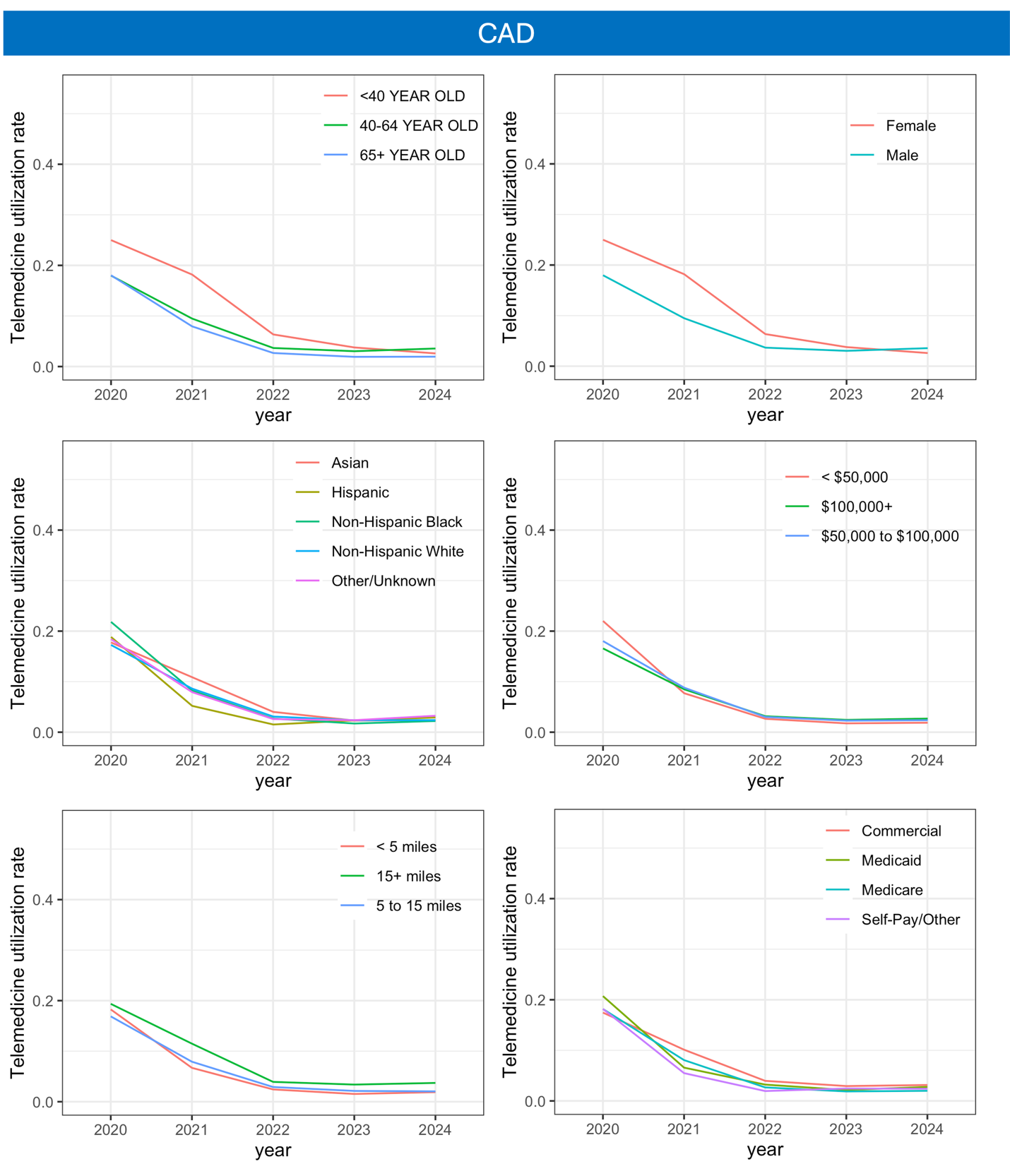


# eFigure 7. Temporal patterns for GI visits


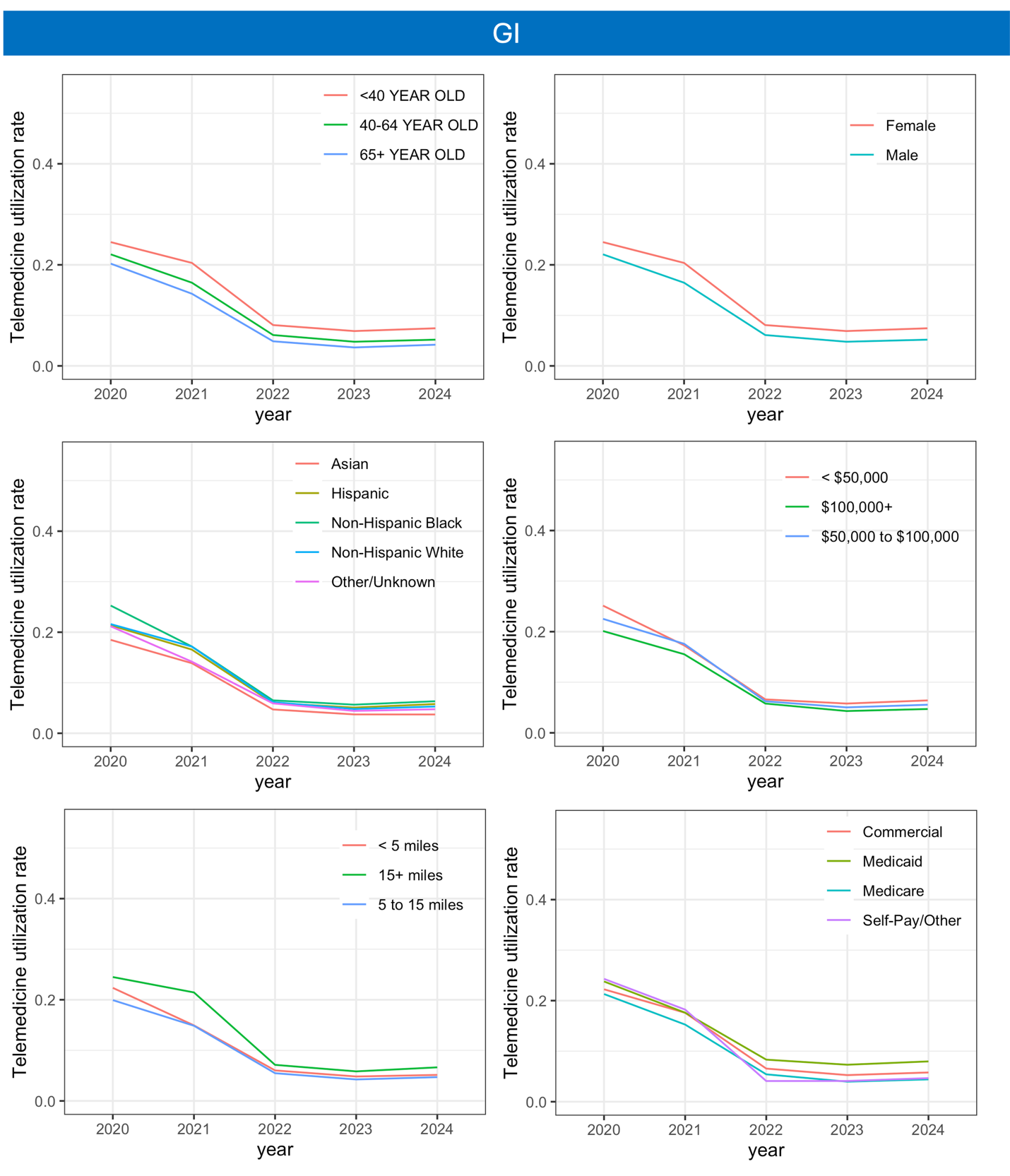

Supplement: Supplementary file 2 — (DOCX 2.65 MB) [file 11606_2025_9964_MOESM2_ESM.docx]
